# Supplementary figures and images for: Rice stripe virus utilizes a Laodelphax striatellus salivary carbonic anhydrase to facilitate plant infection by direct molecular interaction
Source: eLife. 2026 Jan 6;12:RP88132. doi: 10.7554/eLife.88132 (PMC12774414; doi:10.7554/eLife.88132)

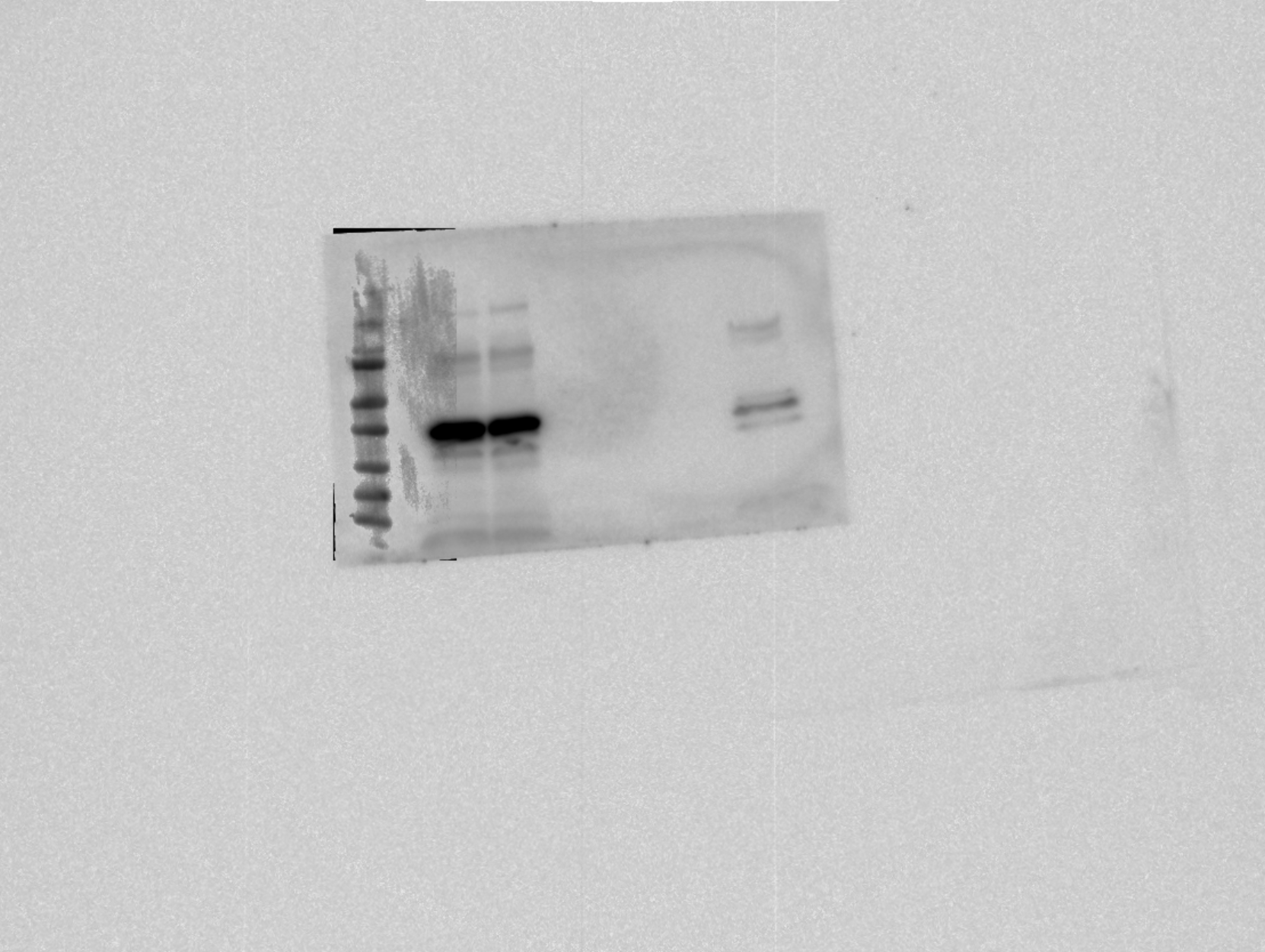

Supplement: Figure 1—source data 1. [file elife-88132-fig1-data1.zip › Figure 1-source data 1/Fig1D/Figure1-D-Blot1-anti-His.tif]

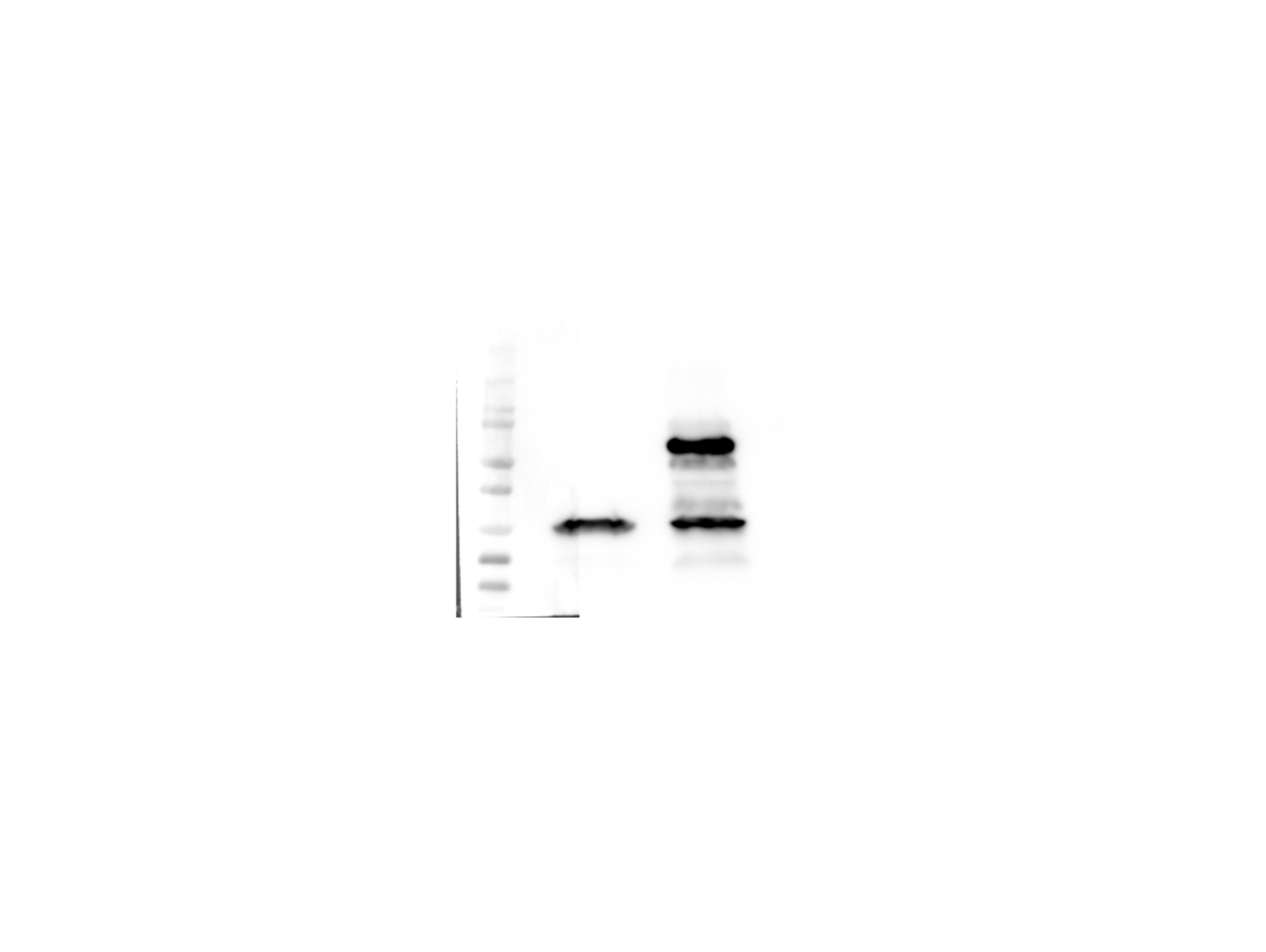

Supplement: Figure 1—source data 1. [file elife-88132-fig1-data1.zip › Figure 1-source data 1/Fig1D/Figure1-D-Blot2-anti-GST.tif]

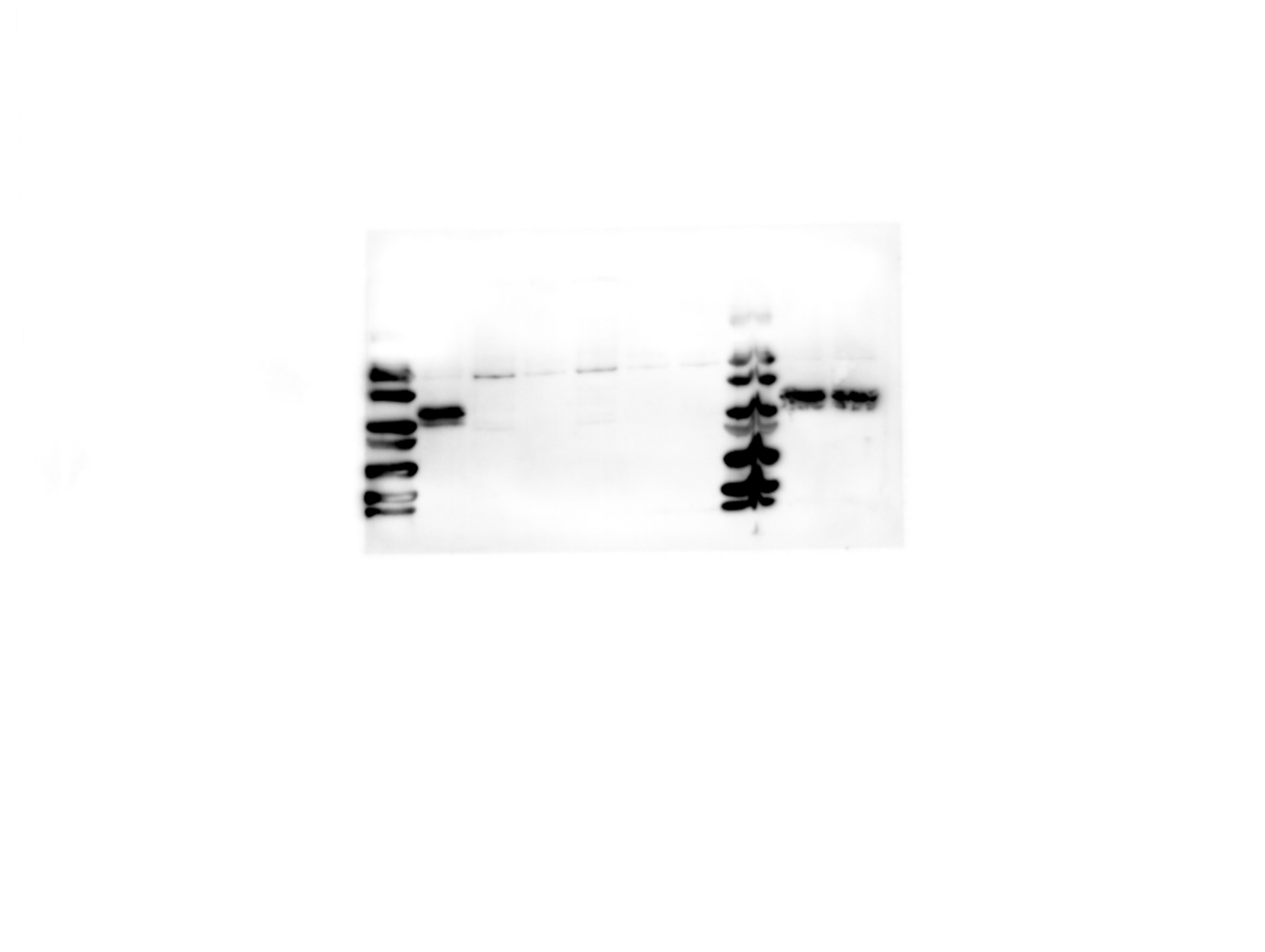

Supplement: Figure 1—source data 1. [file elife-88132-fig1-data1.zip › Figure 1-source data 1/Fig1F/Figure1-F-Blot1-anti-LssaCA.tif]

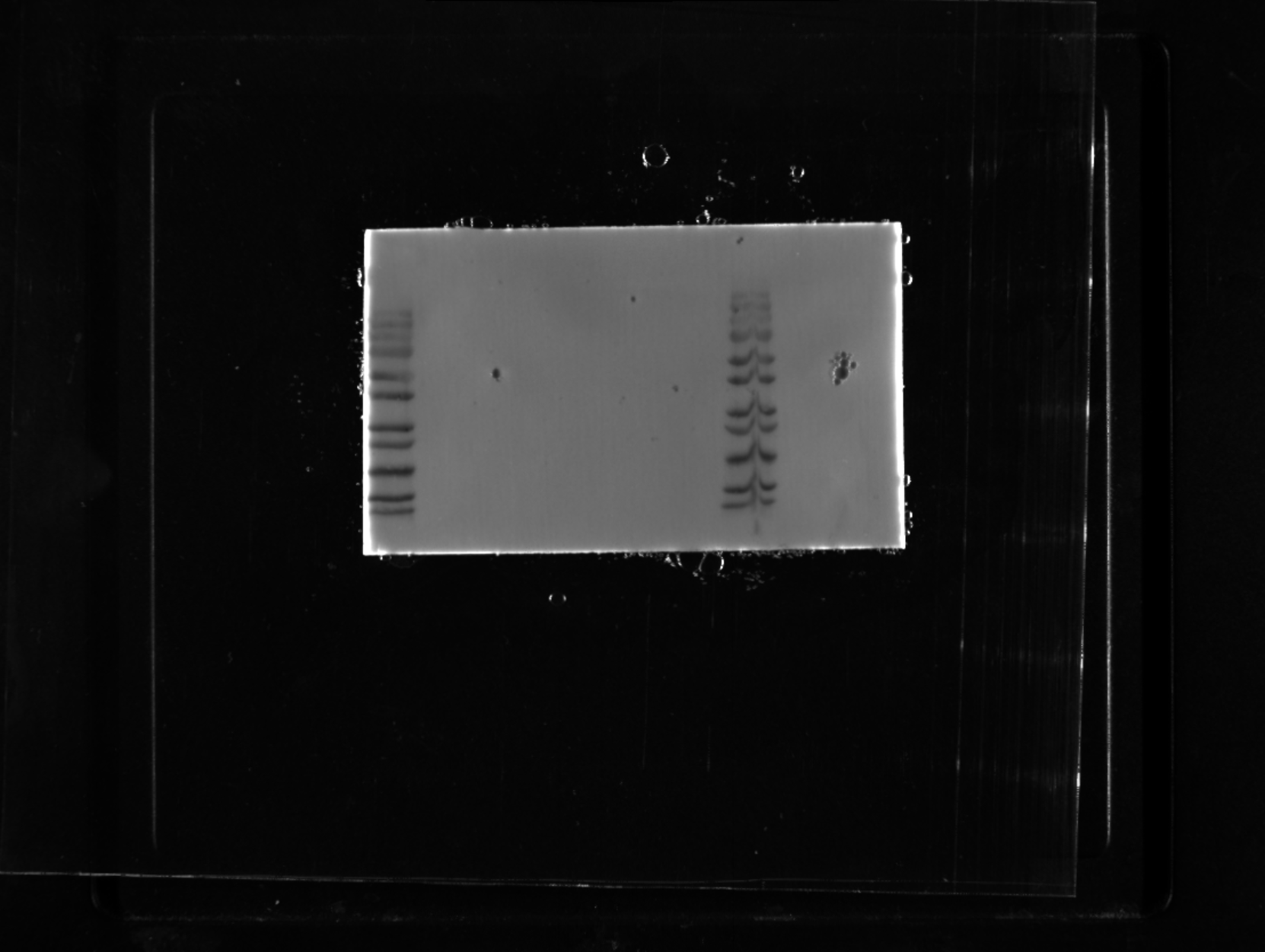

Supplement: Figure 1—source data 1. [file elife-88132-fig1-data1.zip › Figure 1-source data 1/Fig1F/Figure1-F-Blot1-Marker.tif]

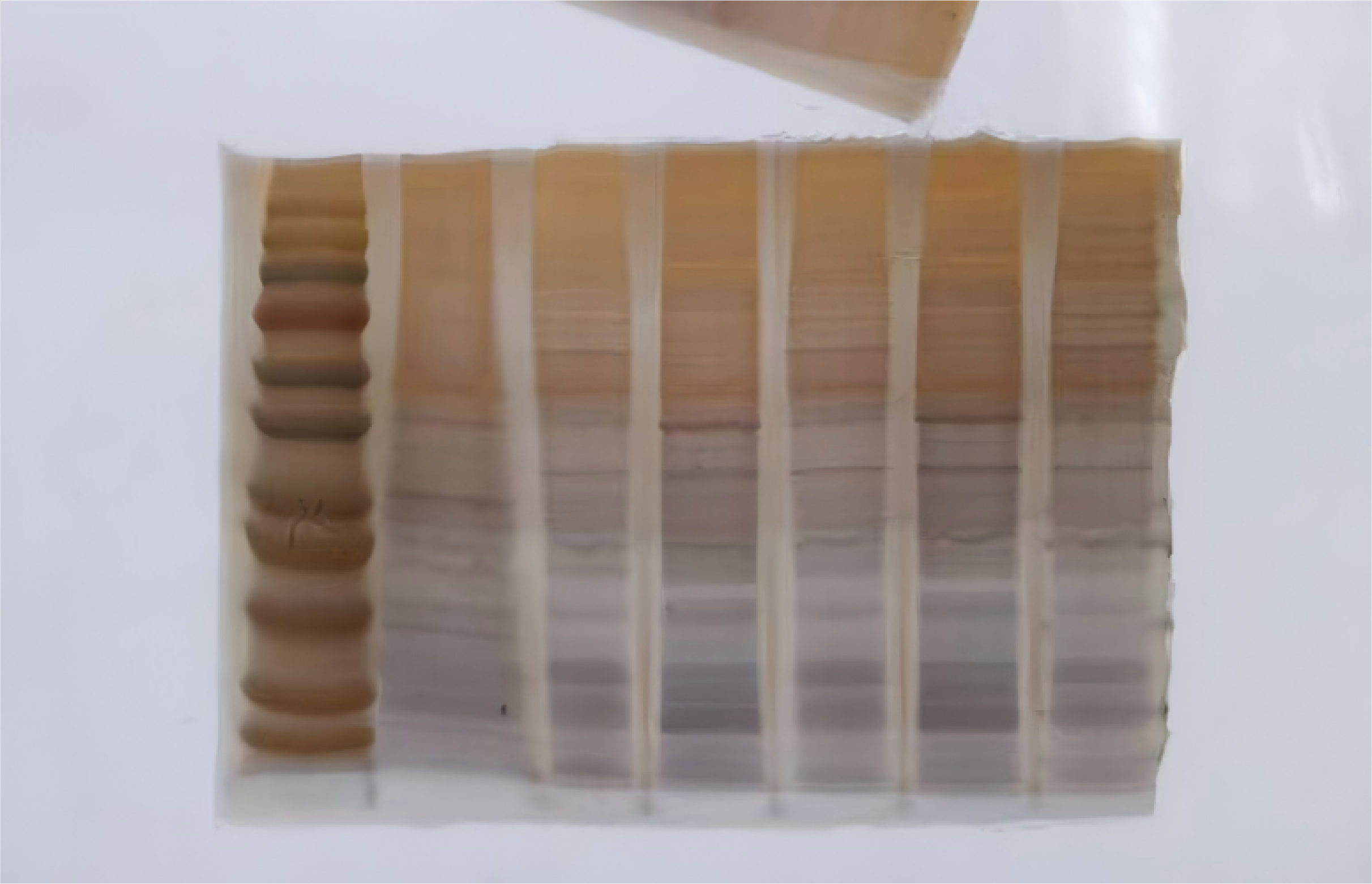

Supplement: Figure 1—source data 1. [file elife-88132-fig1-data1.zip › Figure 1-source data 1/Fig1F/Figure1-F-silver staining.tif]

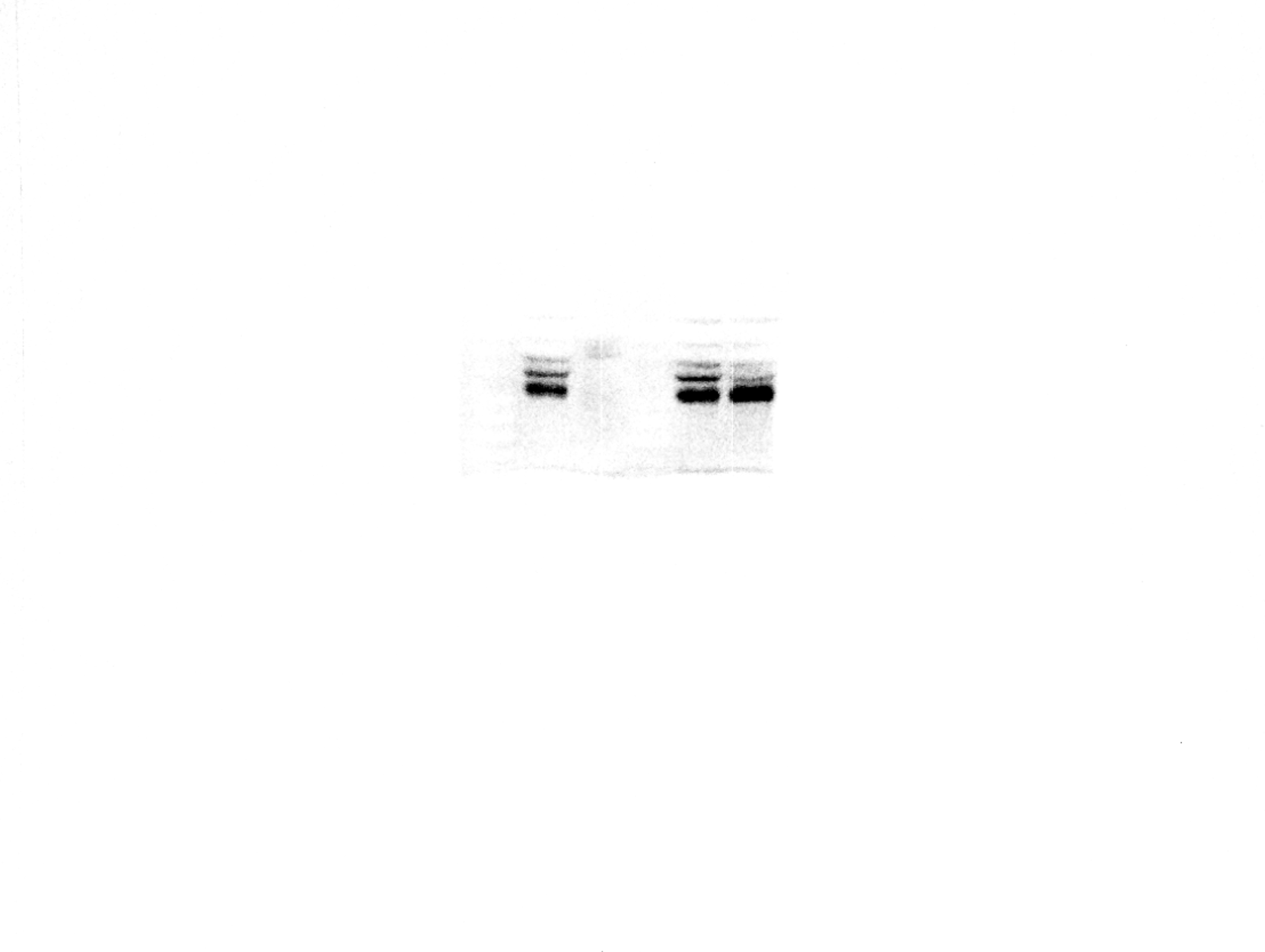

Supplement: Figure 1—source data 1. [file elife-88132-fig1-data1.zip › Figure 1-source data 1/Fig1G/Figure1-G-Blot1-anti-LssaCA.tif]

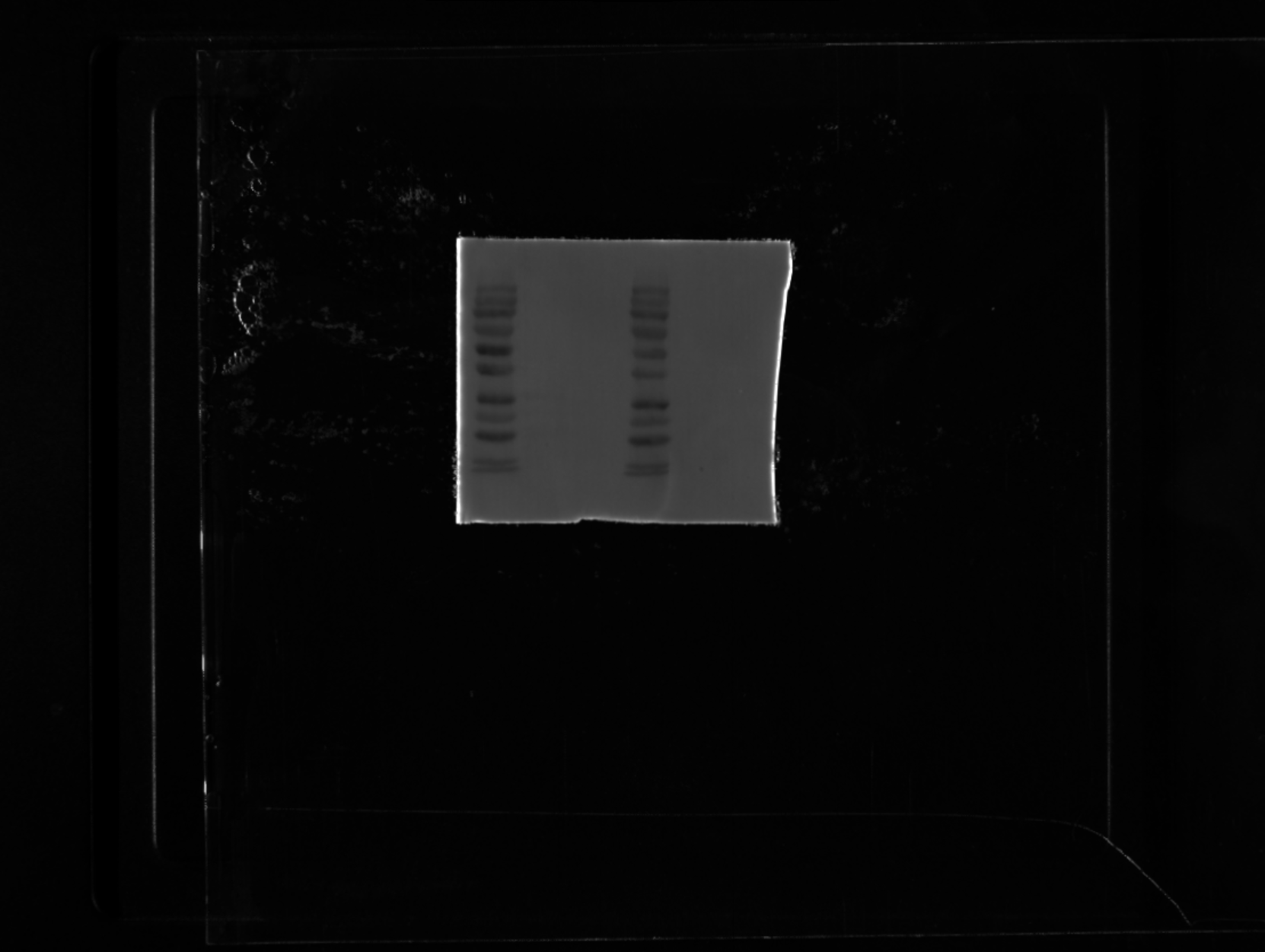

Supplement: Figure 1—source data 1. [file elife-88132-fig1-data1.zip › Figure 1-source data 1/Fig1G/Figure1-G-Blot1-marker.tif]

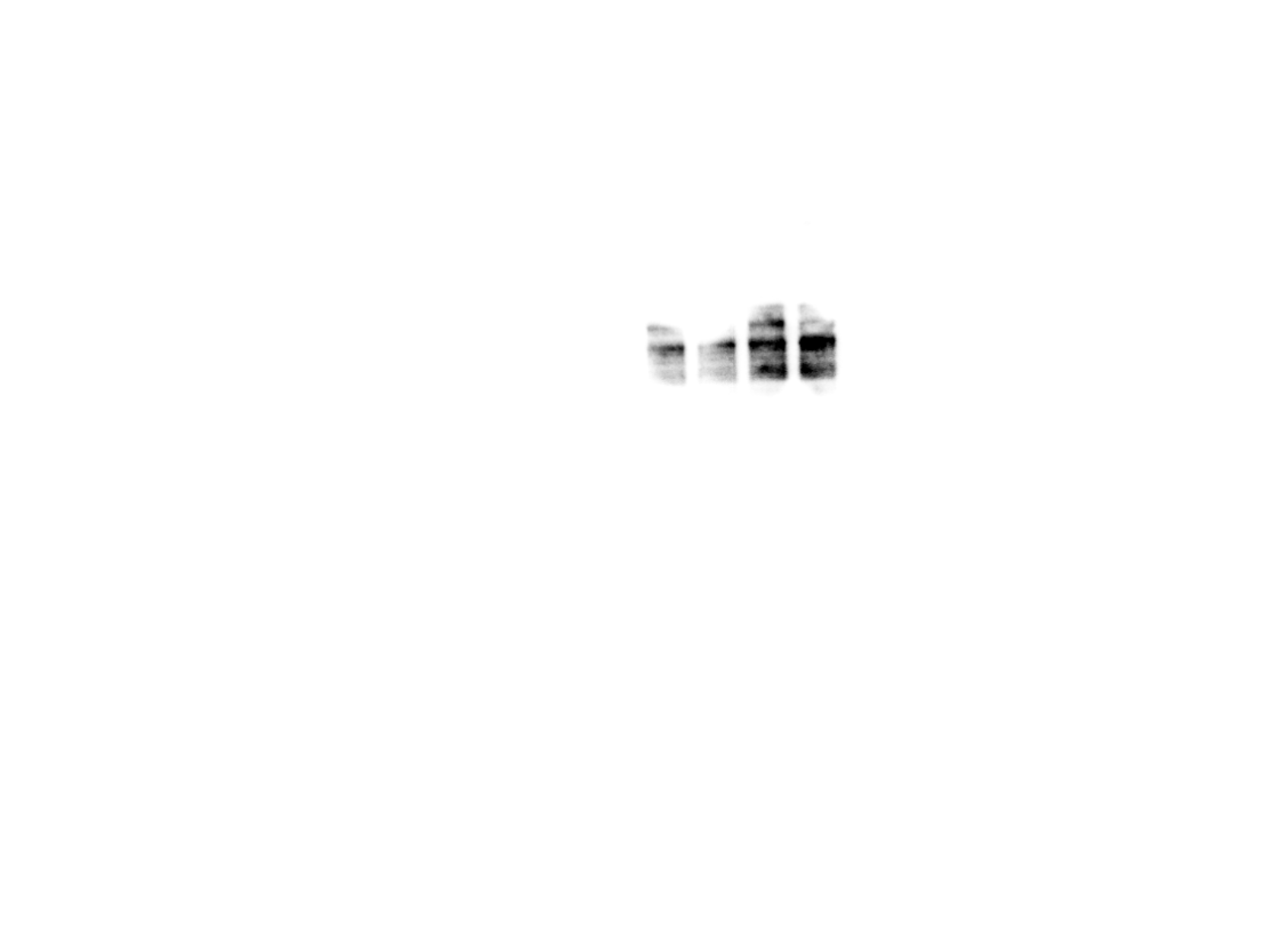

Supplement: Figure 1—source data 1. [file elife-88132-fig1-data1.zip › Figure 1-source data 1/Fig1G/Figure1-G-Blot2-anti-actin.tif]

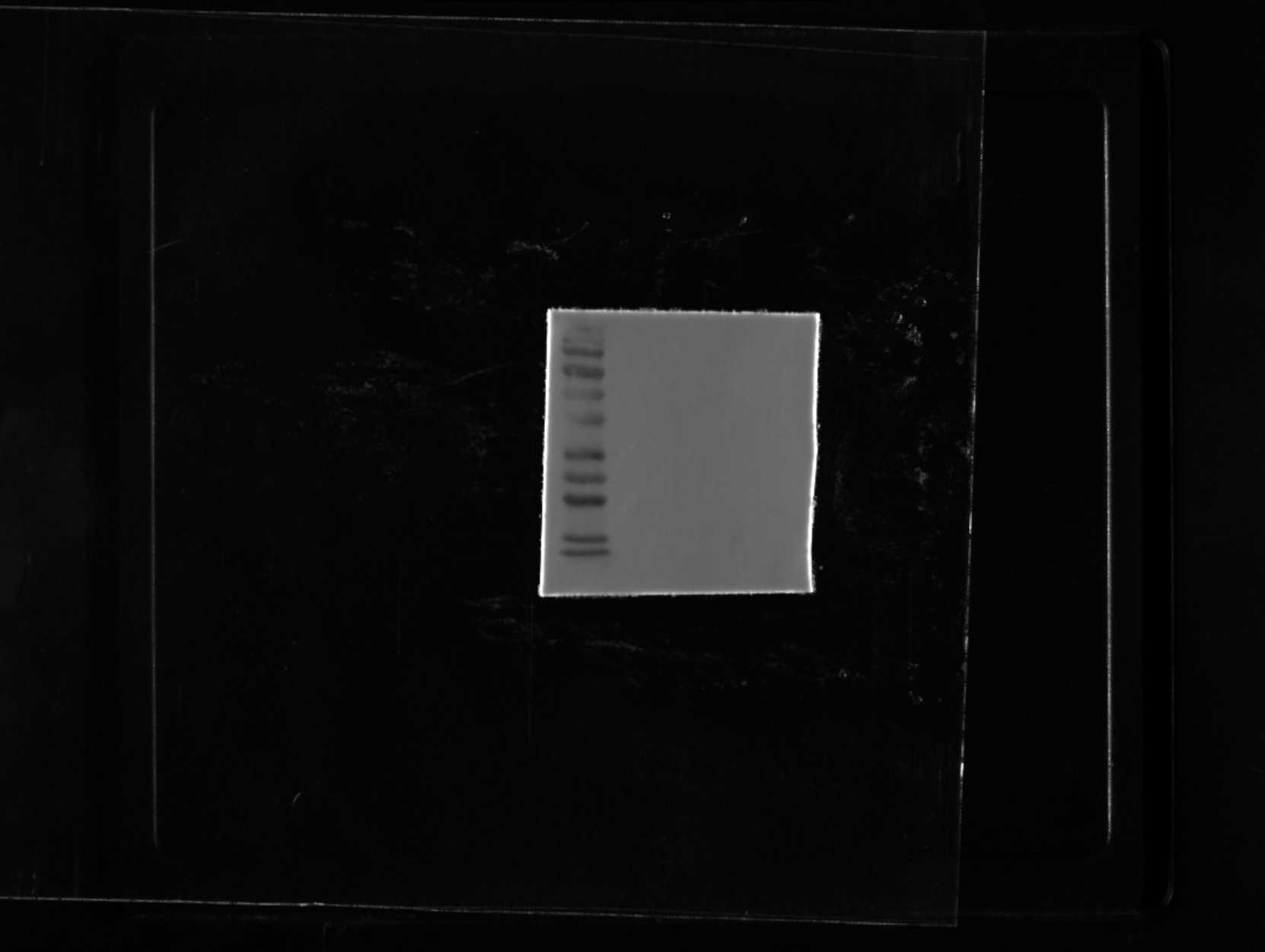

Supplement: Figure 1—source data 1. [file elife-88132-fig1-data1.zip › Figure 1-source data 1/Fig1G/Figure1-G-Blot2-marker.tif]

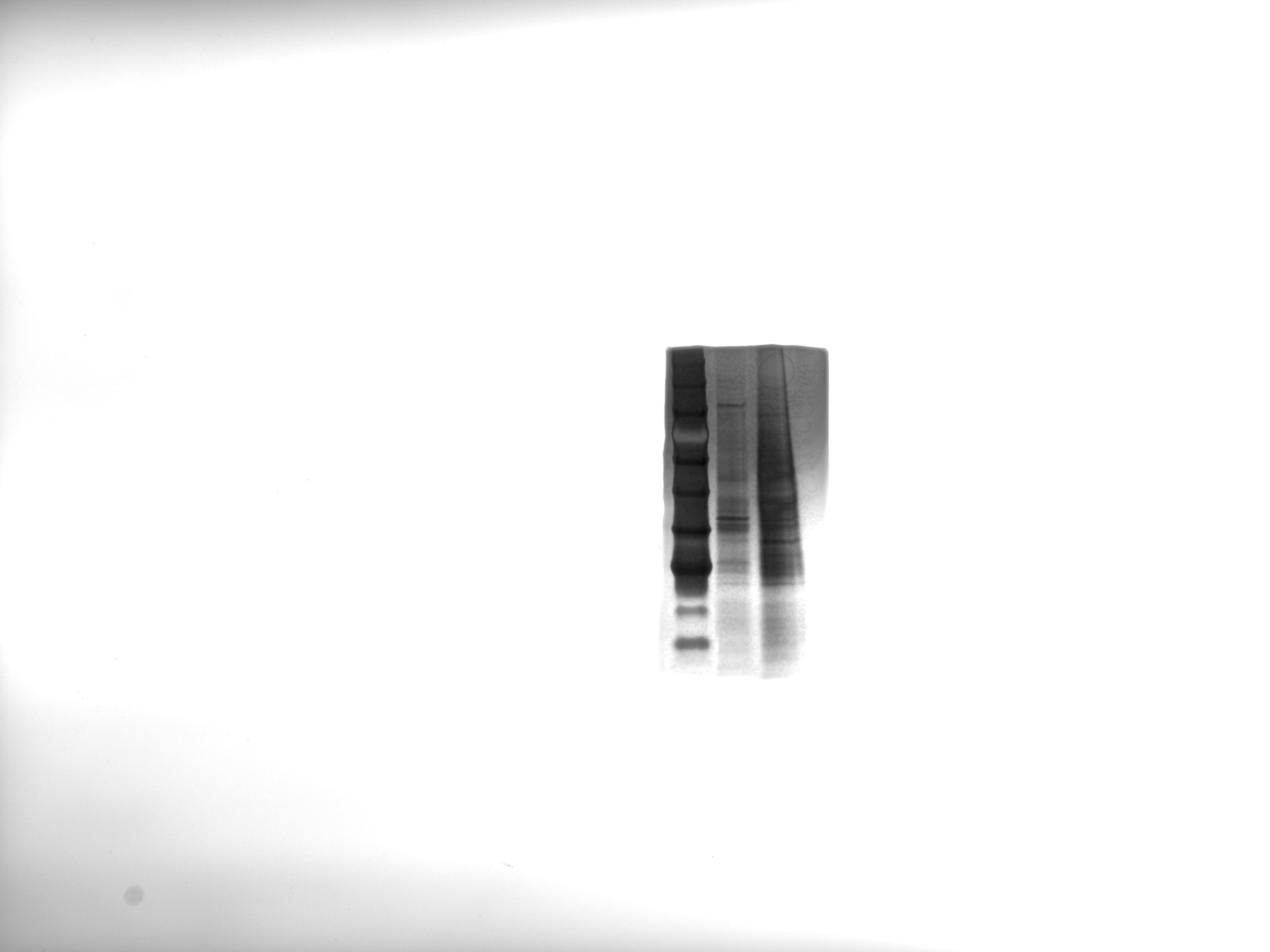

Supplement: Figure 1—source data 1. [file elife-88132-fig1-data1.zip › Figure 1-source data 1/Fig1H/Figure-H-silver staining.tif]

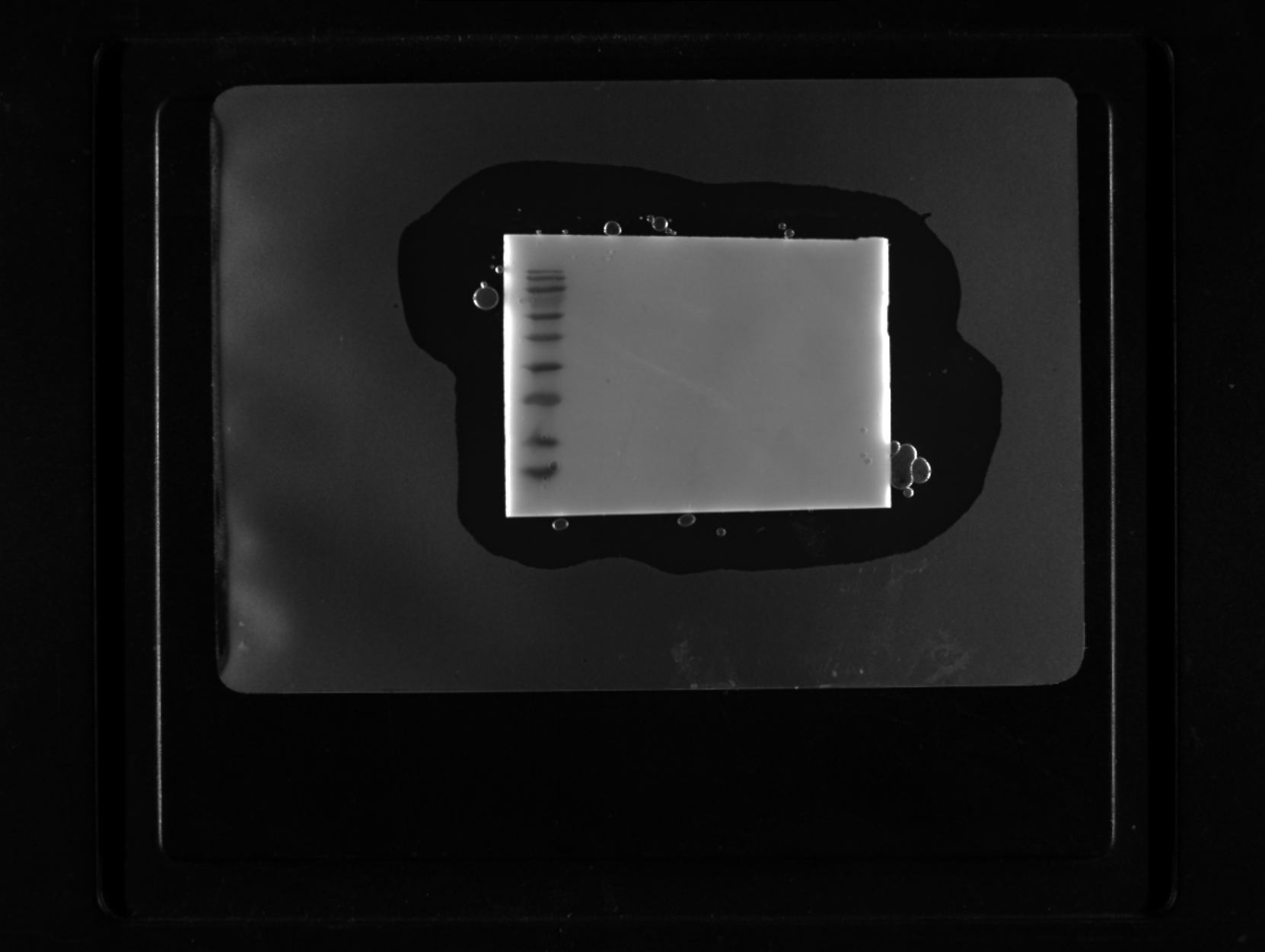

Supplement: Figure 1—source data 1. [file elife-88132-fig1-data1.zip › Figure 1-source data 1/Fig1H/Figure1-H-Blot1-marker.tif]

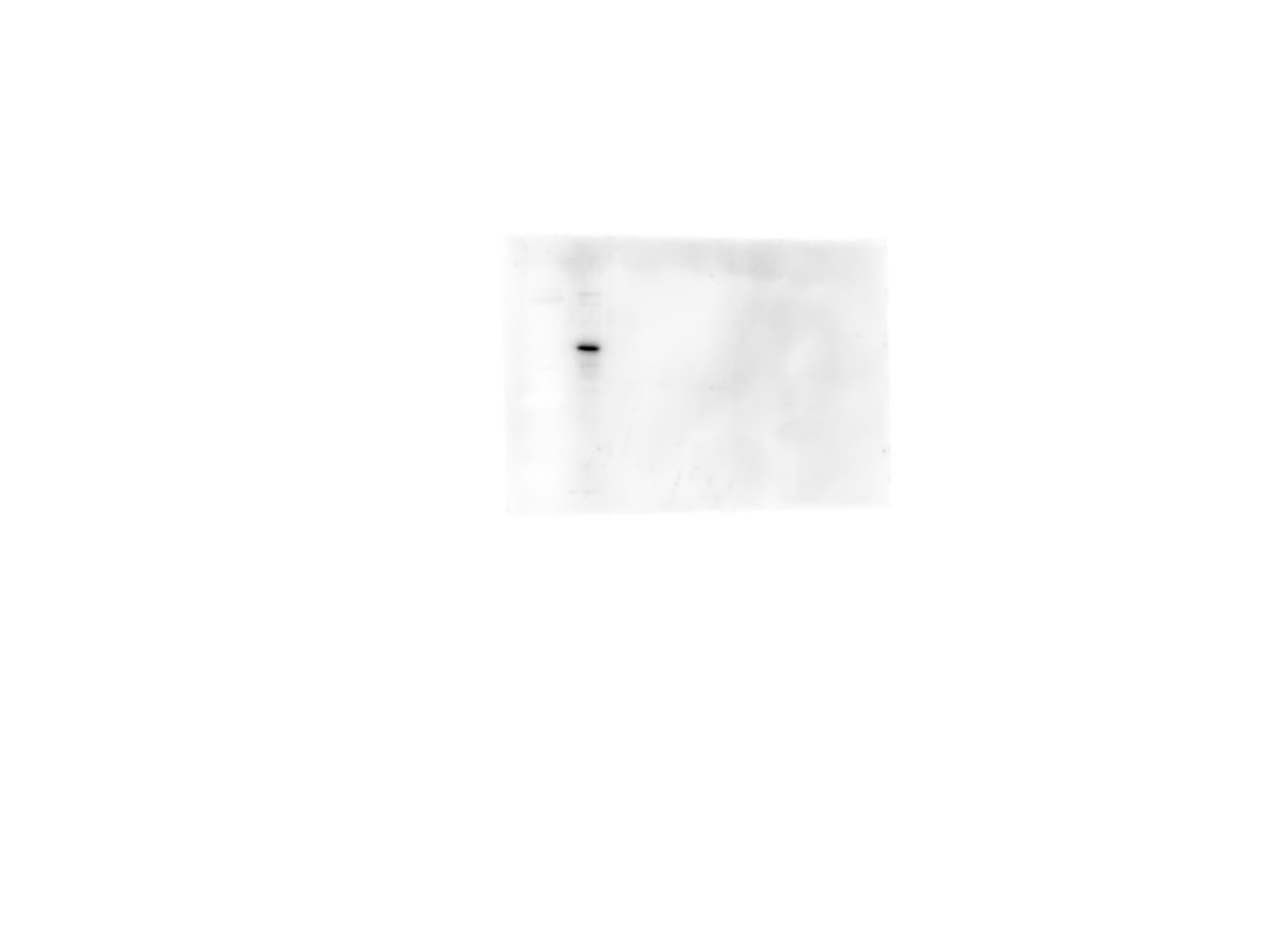

Supplement: Figure 1—source data 1. [file elife-88132-fig1-data1.zip › Figure 1-source data 1/Fig1H/Figure1-H-Blot2-anti-LssaCA.tif]

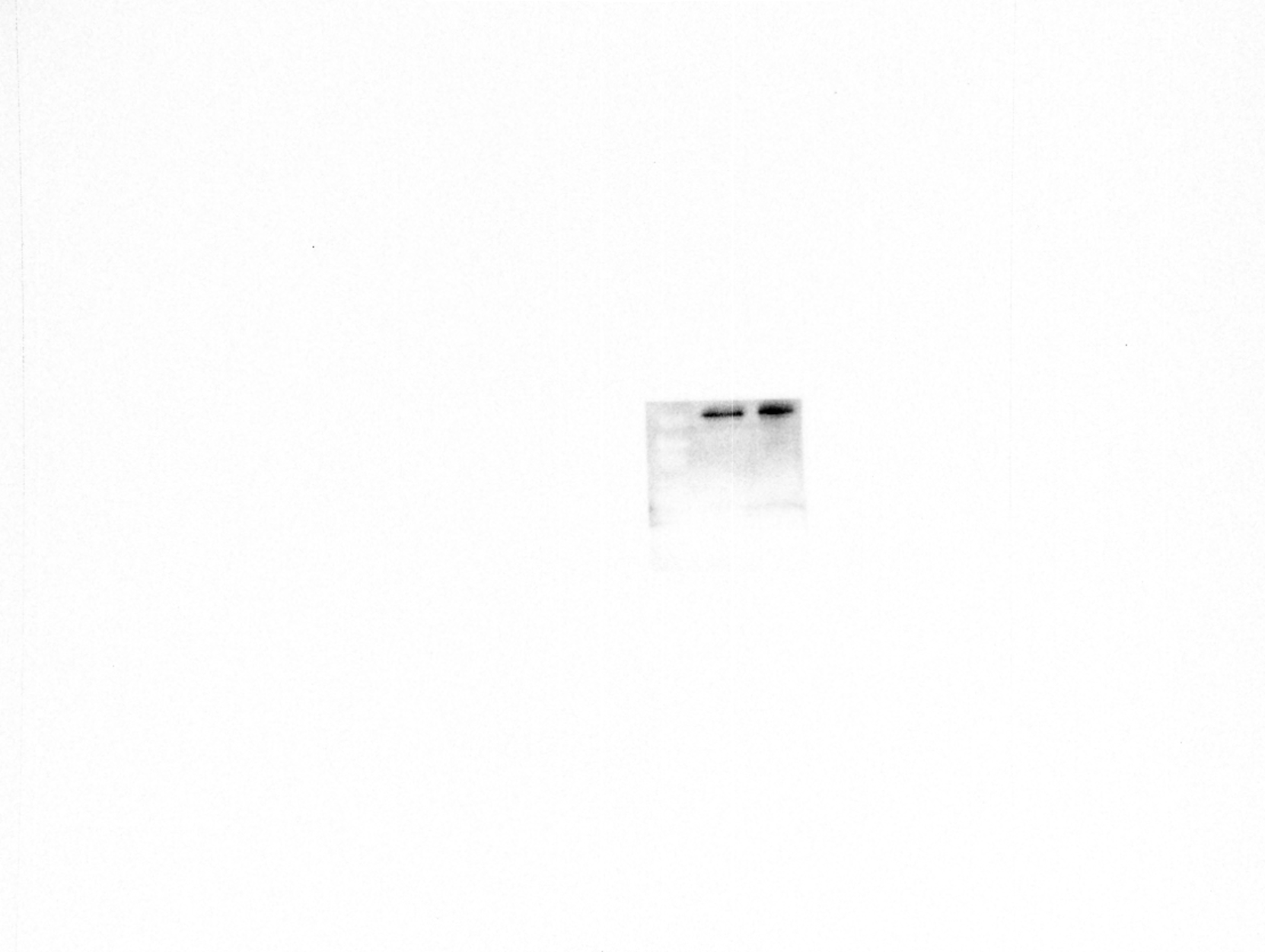

Supplement: Figure 1—source data 1. [file elife-88132-fig1-data1.zip › Figure 1-source data 1/Fig1I/Figure1-I-Blot1-anti-LssaCA.tif]

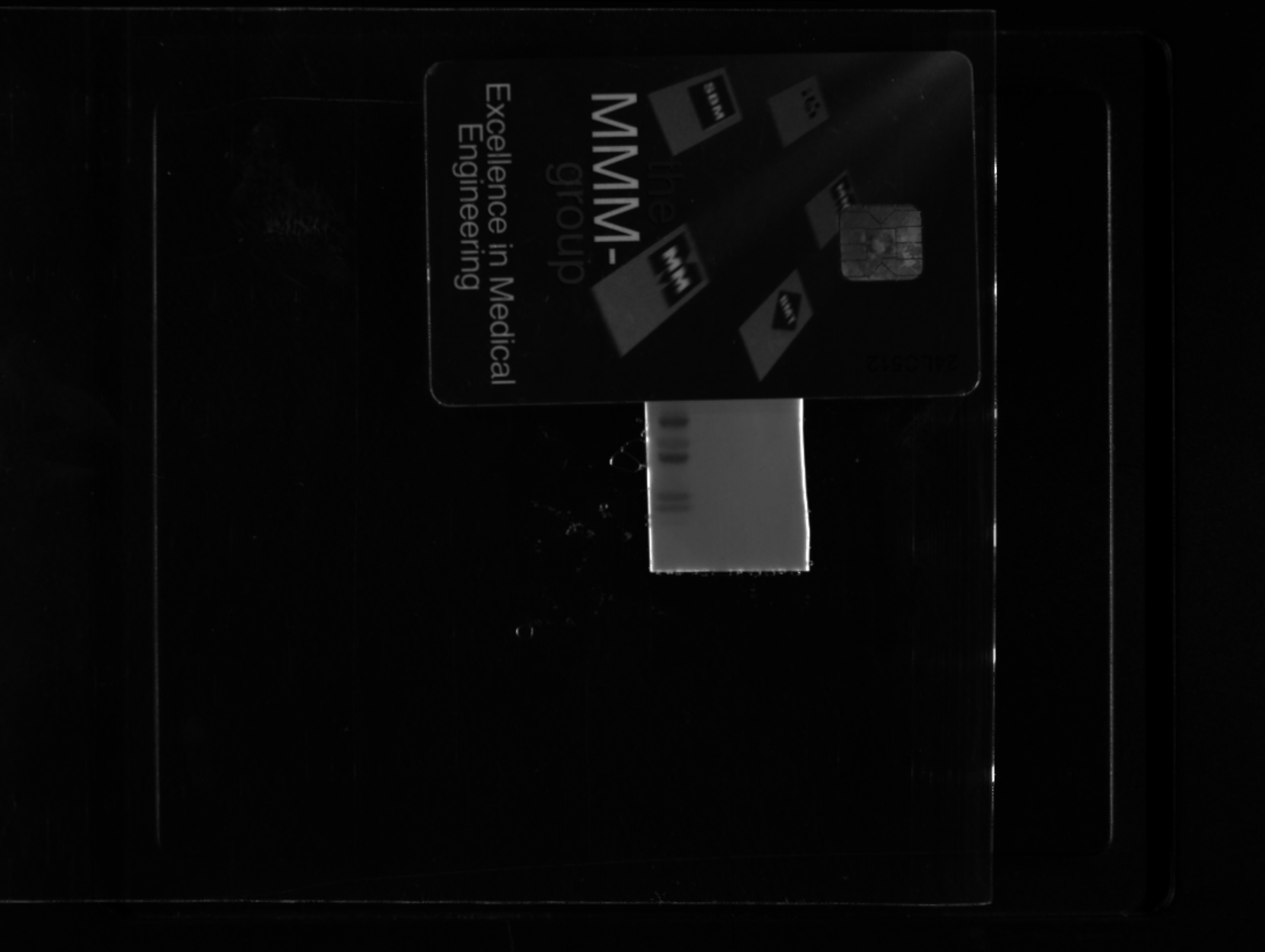

Supplement: Figure 1—source data 1. [file elife-88132-fig1-data1.zip › Figure 1-source data 1/Fig1I/Figure1-I-Blot1-marker.tif]

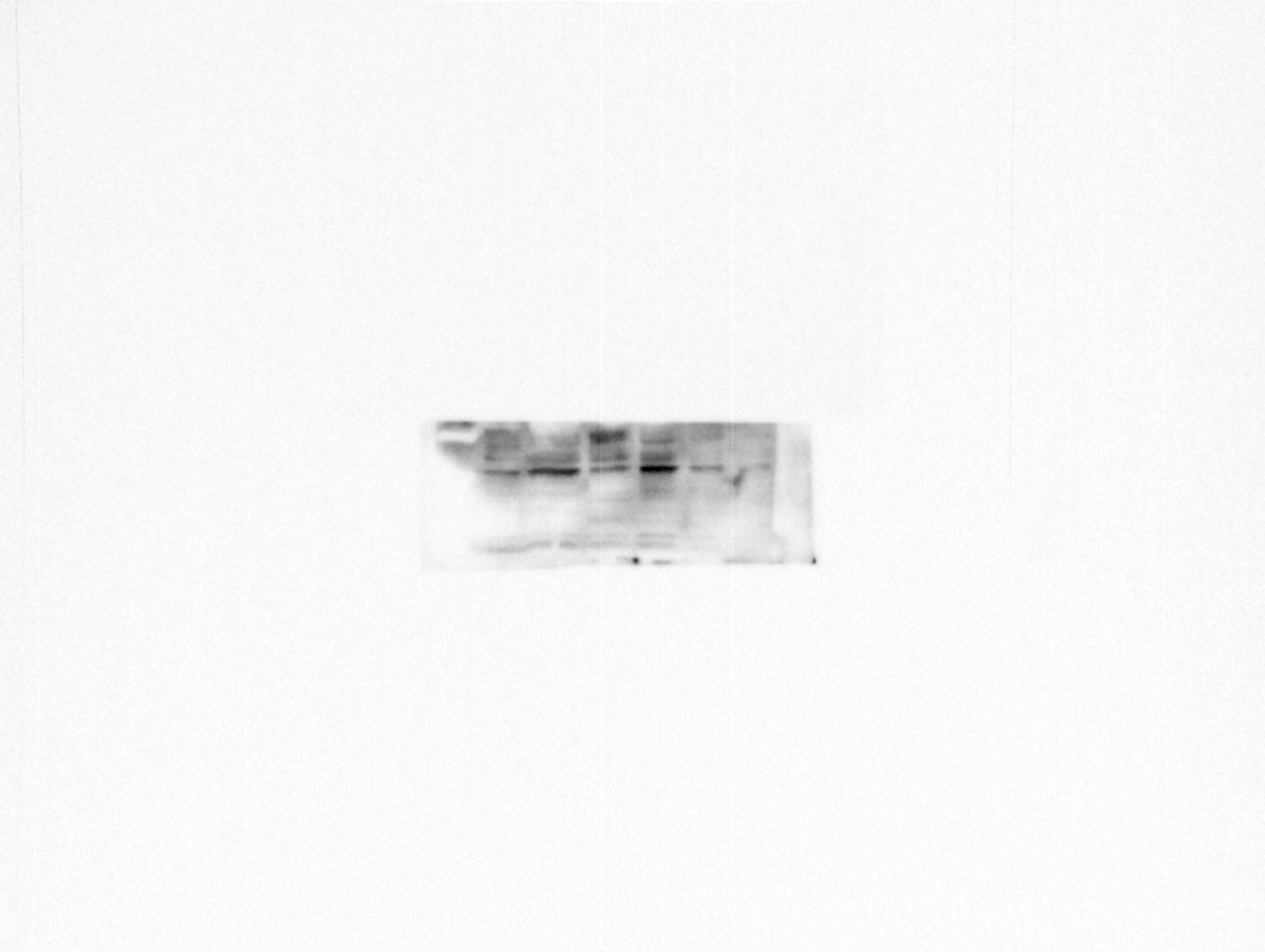

Supplement: Figure 1—source data 1. [file elife-88132-fig1-data1.zip › Figure 1-source data 1/Fig1I/Figure1-I-Blot2-anti-LssaCA.tif]

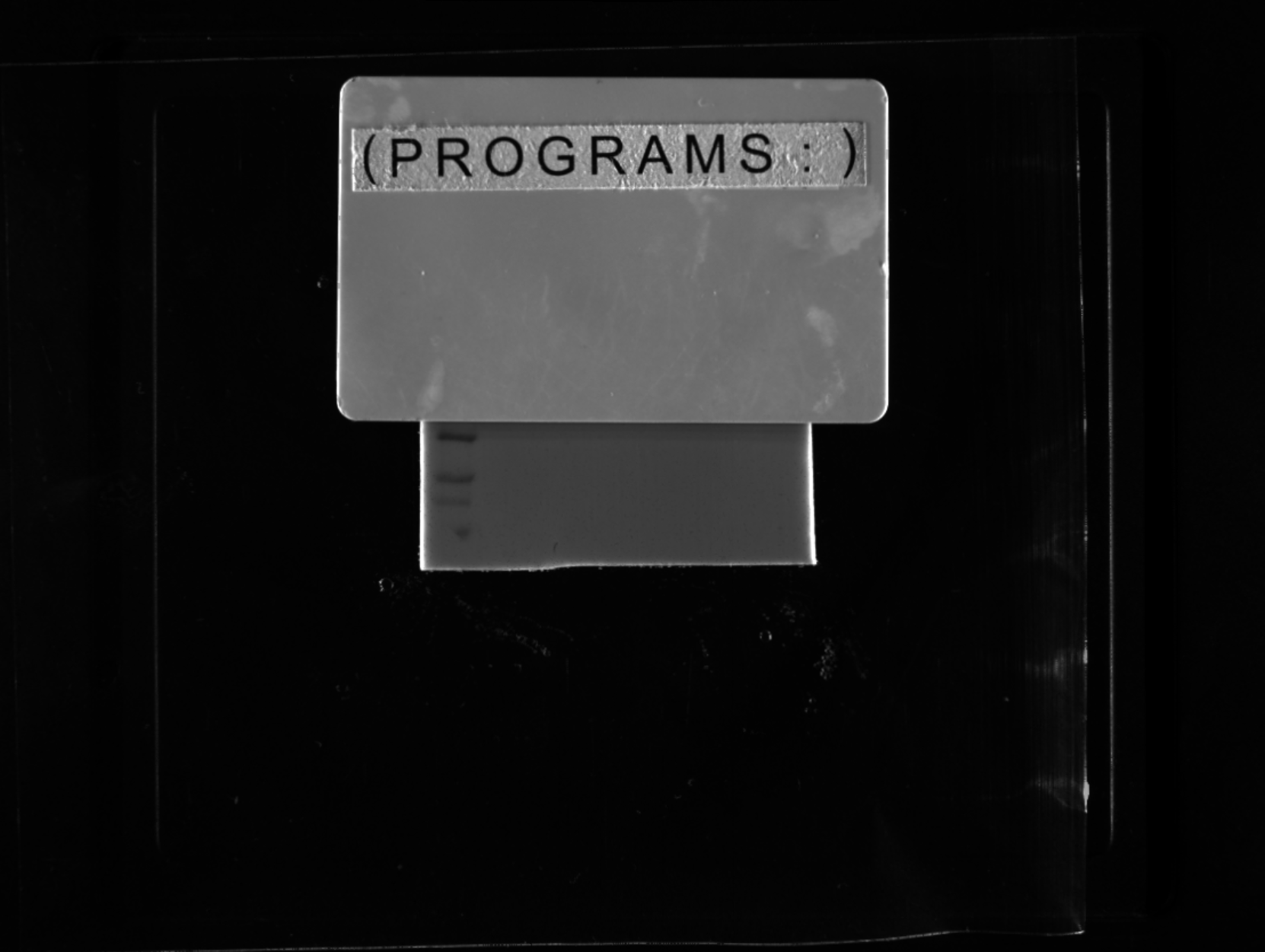

Supplement: Figure 1—source data 1. [file elife-88132-fig1-data1.zip › Figure 1-source data 1/Fig1I/Figure1-I-Blot2-marker.tif]

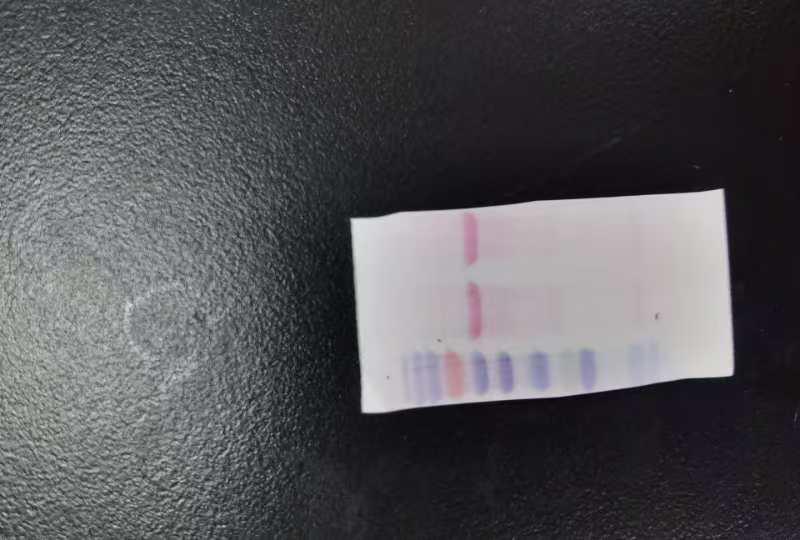

Supplement: Figure 1—source data 1. [file elife-88132-fig1-data1.zip › Figure 1-source data 1/Fig1I/Figure1-I-Ponceau S1-RBCL.jpg]

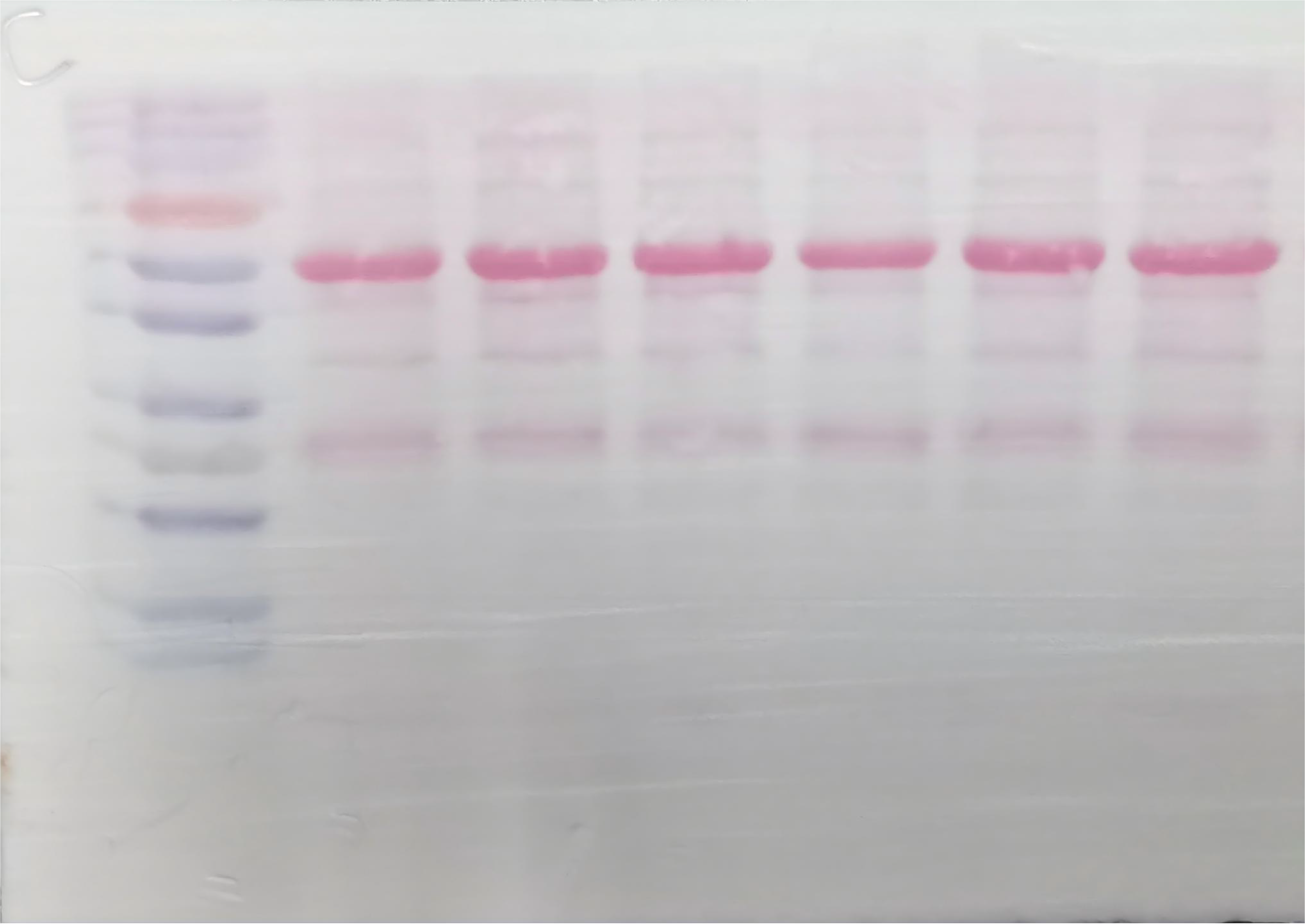

Supplement: Figure 1—source data 1. [file elife-88132-fig1-data1.zip › Figure 1-source data 1/Fig1I/Figure1-I-Ponceau S2-RBCL.tif]

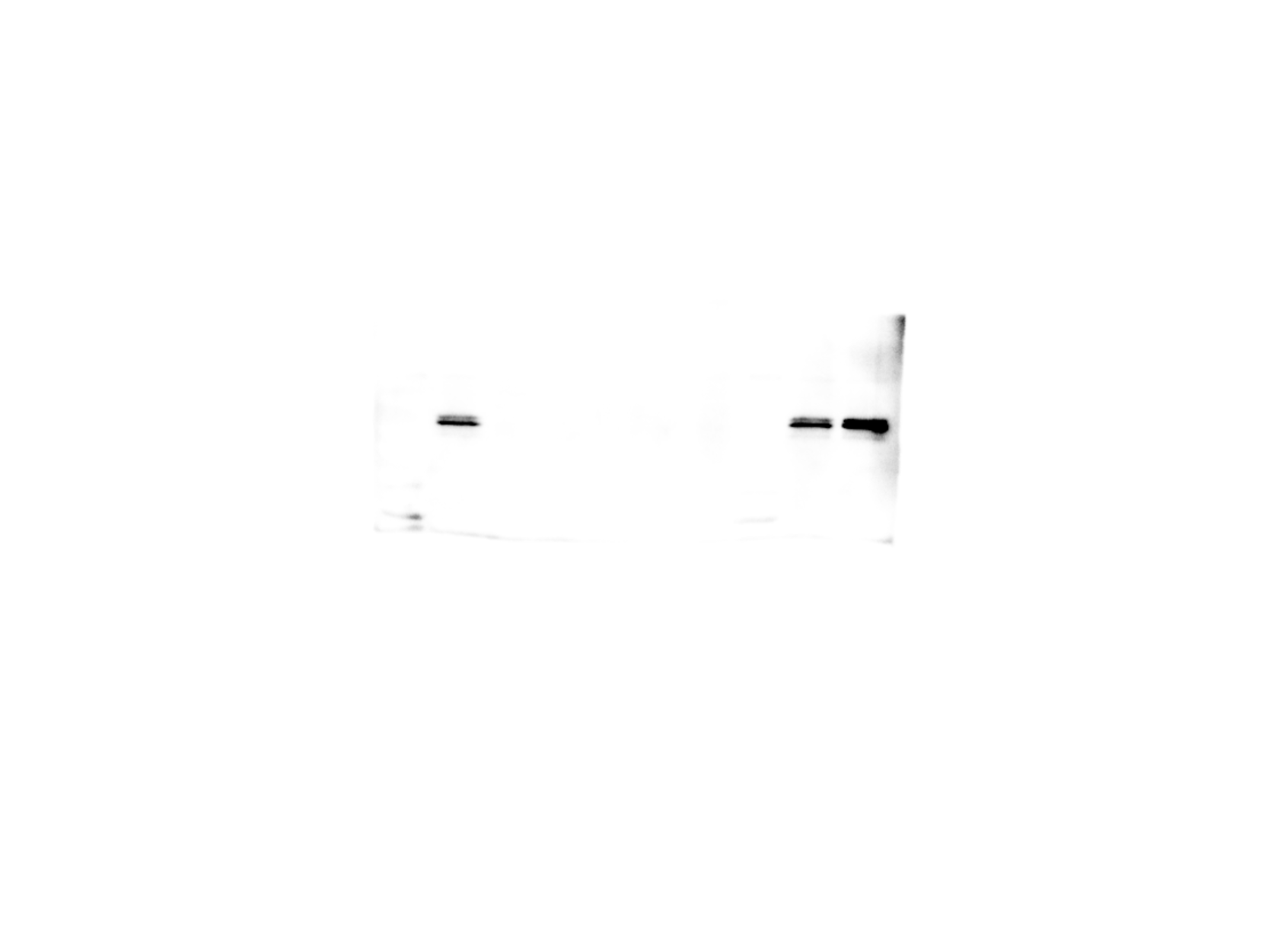

Supplement: Figure 1—source data 1. [file elife-88132-fig1-data1.zip › Figure 1-source data 1/Fig1J/Figure1-J-Blot1-anti-LssaCA.tif]

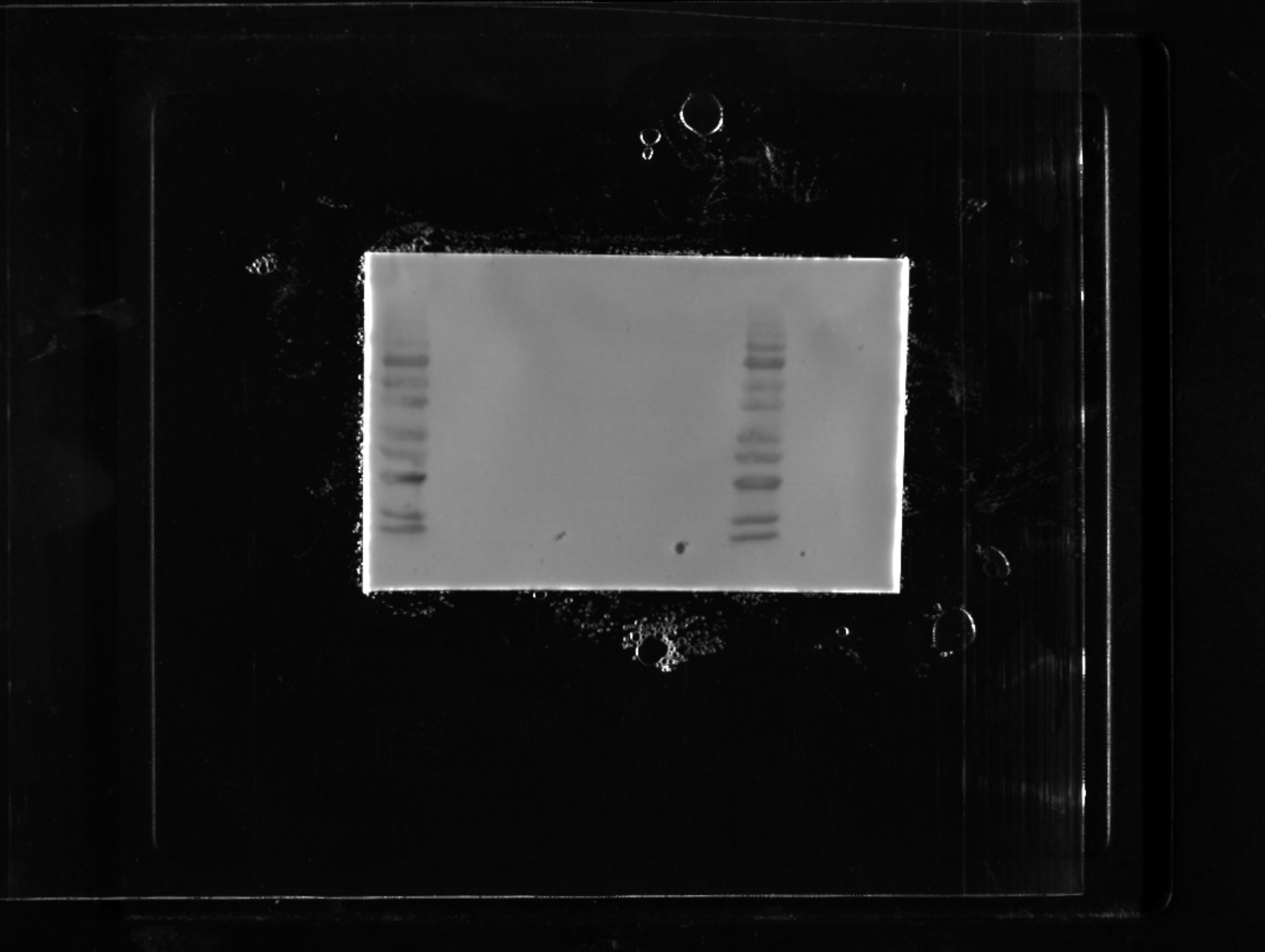

Supplement: Figure 1—source data 1. [file elife-88132-fig1-data1.zip › Figure 1-source data 1/Fig1J/Figure1-J-Blot1-marker.tif]

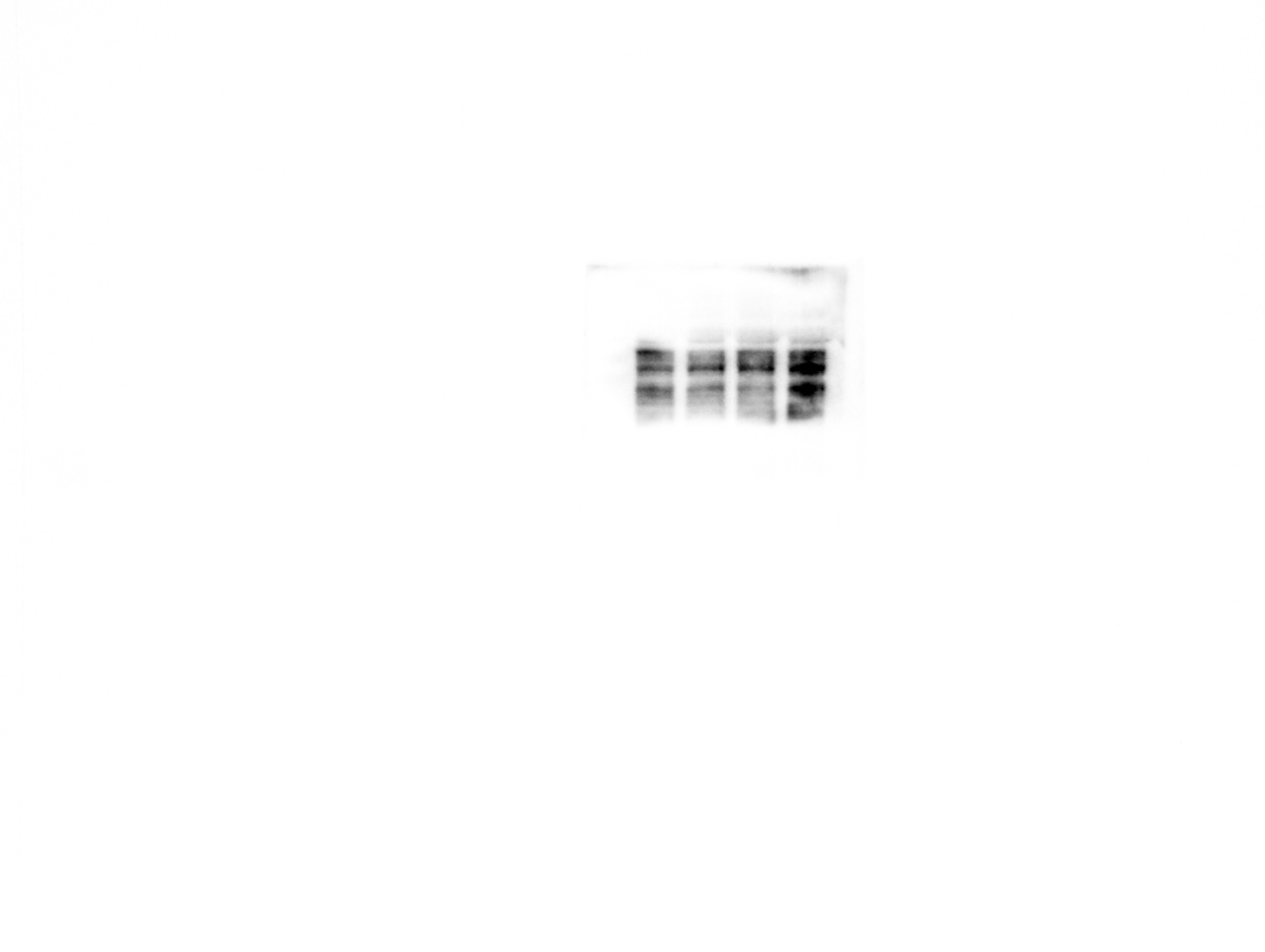

Supplement: Figure 1—source data 1. [file elife-88132-fig1-data1.zip › Figure 1-source data 1/Fig1J/Figure1-J-Blot2-anti-actin.tif]

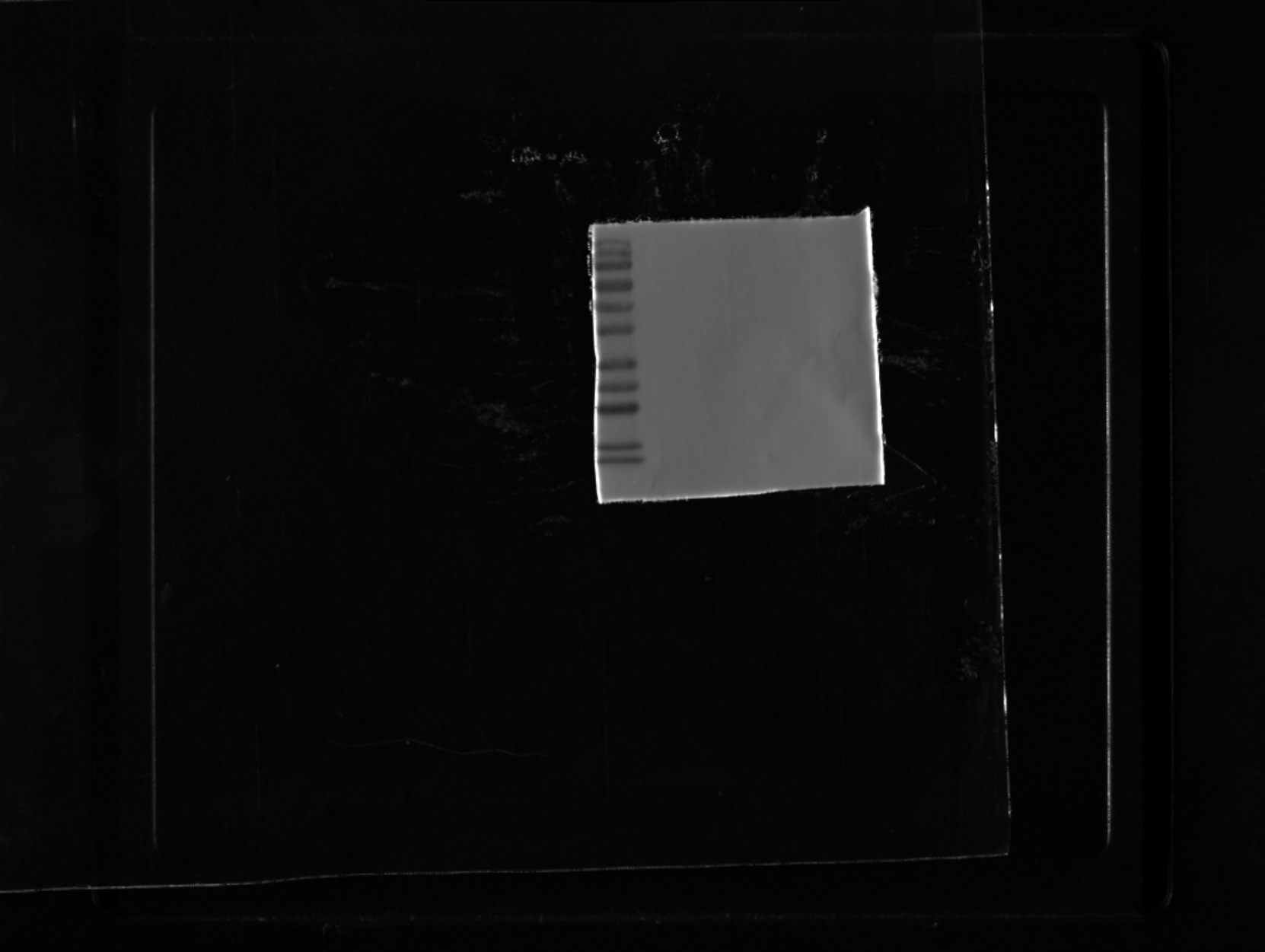

Supplement: Figure 1—source data 1. [file elife-88132-fig1-data1.zip › Figure 1-source data 1/Fig1J/Figure1-J-Blot2-marker.tif]

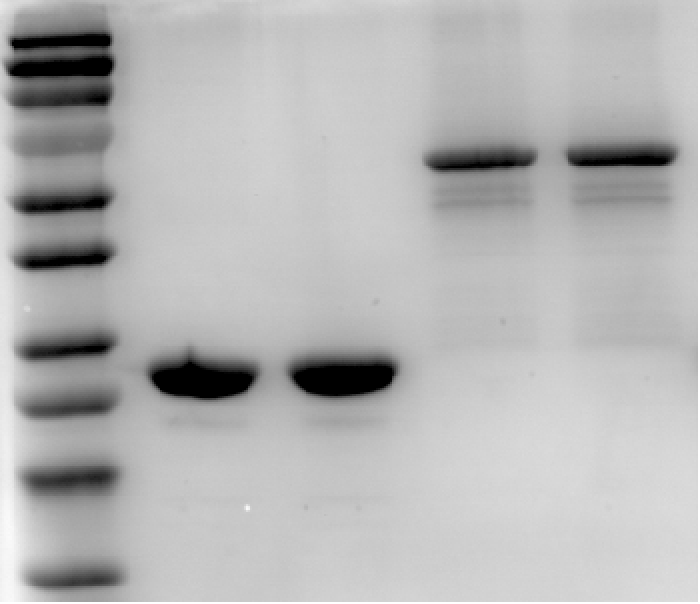

Supplement: Figure 1—figure supplement 3—source data 1. [file elife-88132-fig1-figsupp3-data1.zip › Figure 1-figure supplement 3-source data 1/Figure1-supplement3-gal1.tif]

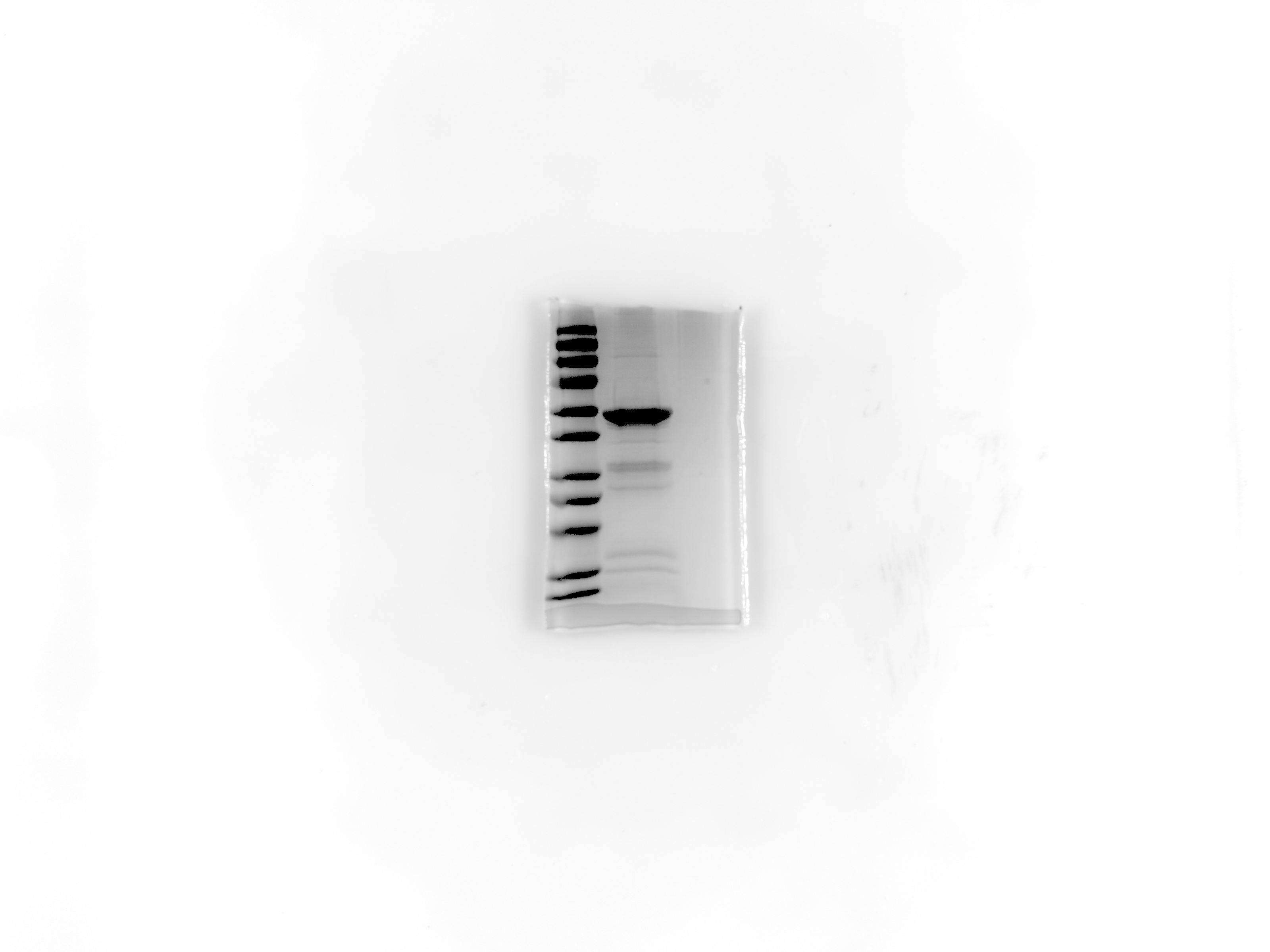

Supplement: Figure 1—figure supplement 3—source data 1. [file elife-88132-fig1-figsupp3-data1.zip › Figure 1-figure supplement 3-source data 1/Figure1-supplement3-gal2.tif]

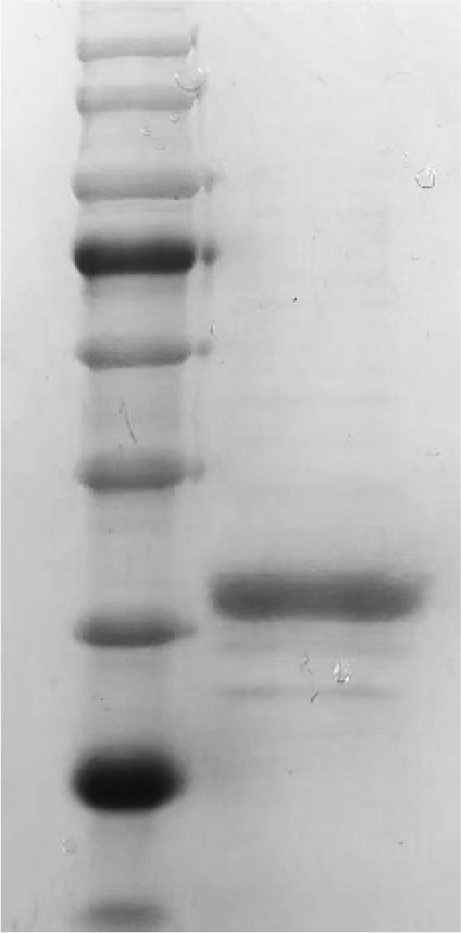

Supplement: Figure 1—figure supplement 3—source data 1. [file elife-88132-fig1-figsupp3-data1.zip › Figure 1-figure supplement 3-source data 1/Figure1-supplement3-gal3.tif]

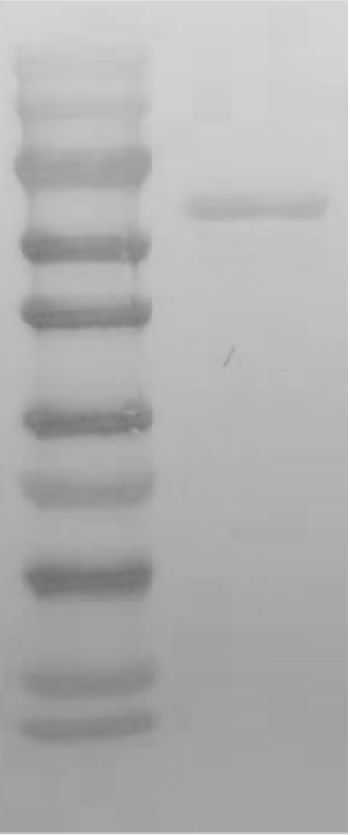

Supplement: Figure 1—figure supplement 3—source data 1. [file elife-88132-fig1-figsupp3-data1.zip › Figure 1-figure supplement 3-source data 1/Figure1-supplement3-gal4.tif]

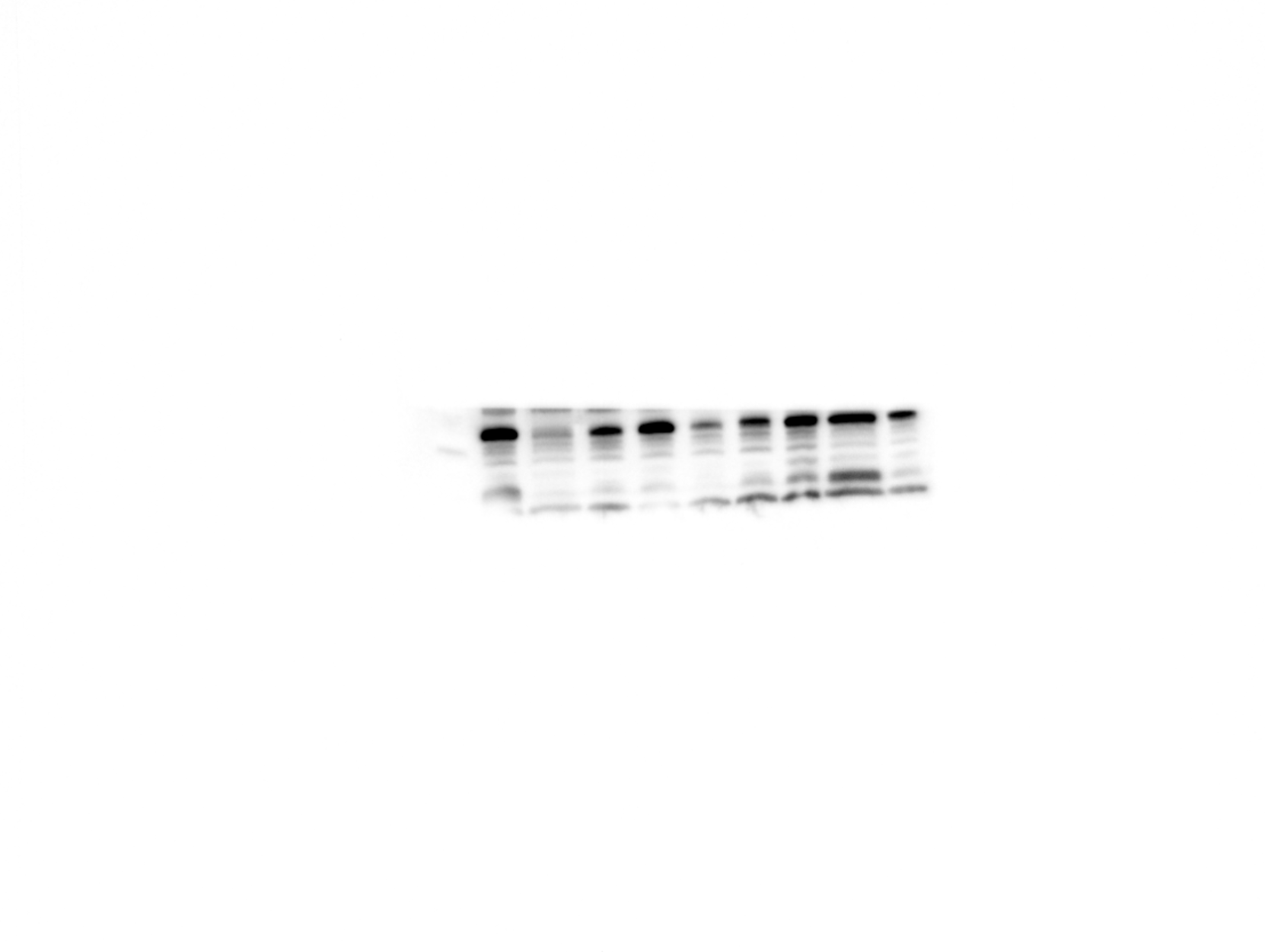

Supplement: Figure 2—source data 1. [file elife-88132-fig2-data1.zip › Figure 2-source data 1/Fig2C/Figure2-C-Blot1-anti-NP.tif]

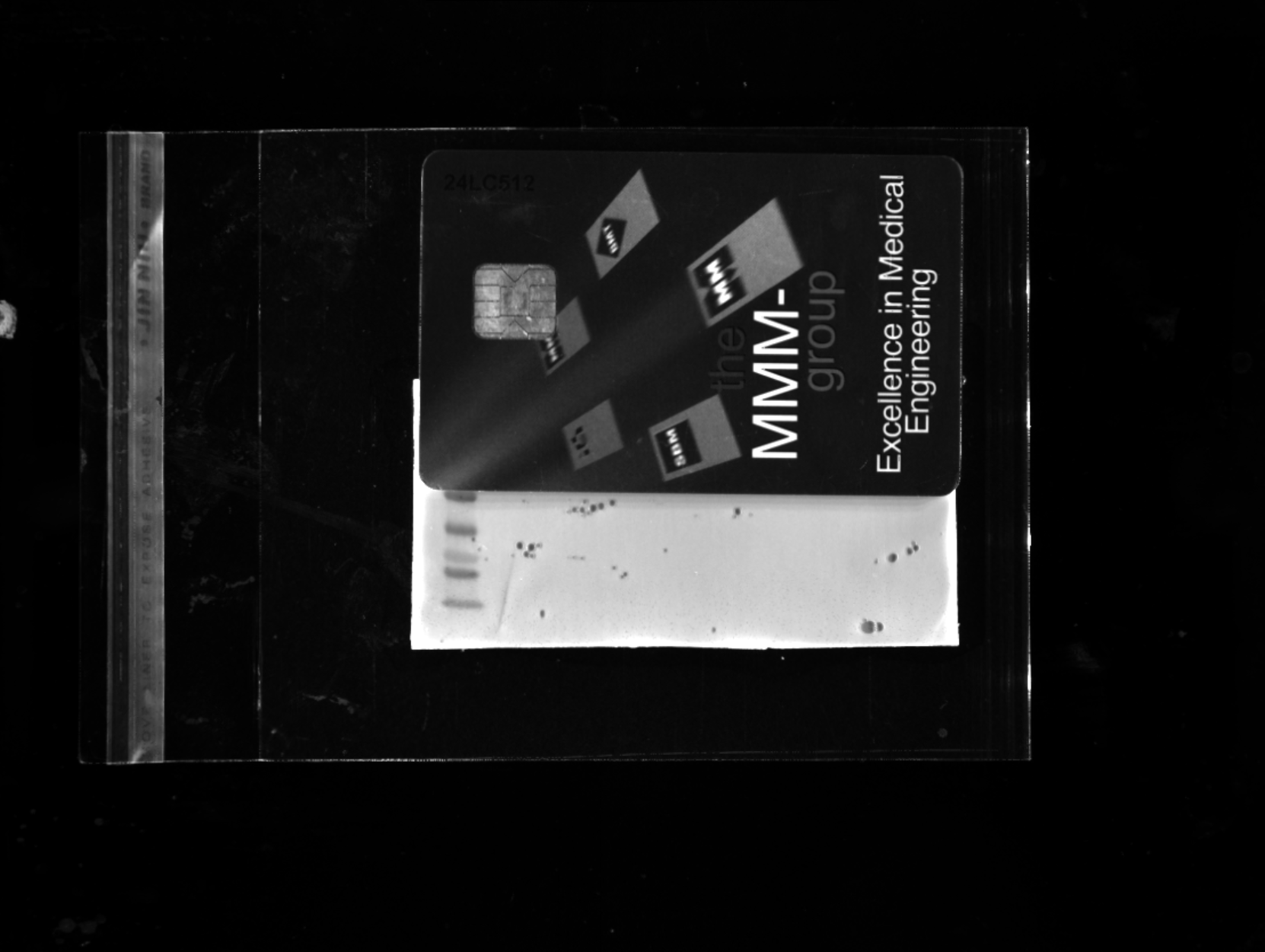

Supplement: Figure 2—source data 1. [file elife-88132-fig2-data1.zip › Figure 2-source data 1/Fig2C/Figure2-C-Blot1-marker.tif]

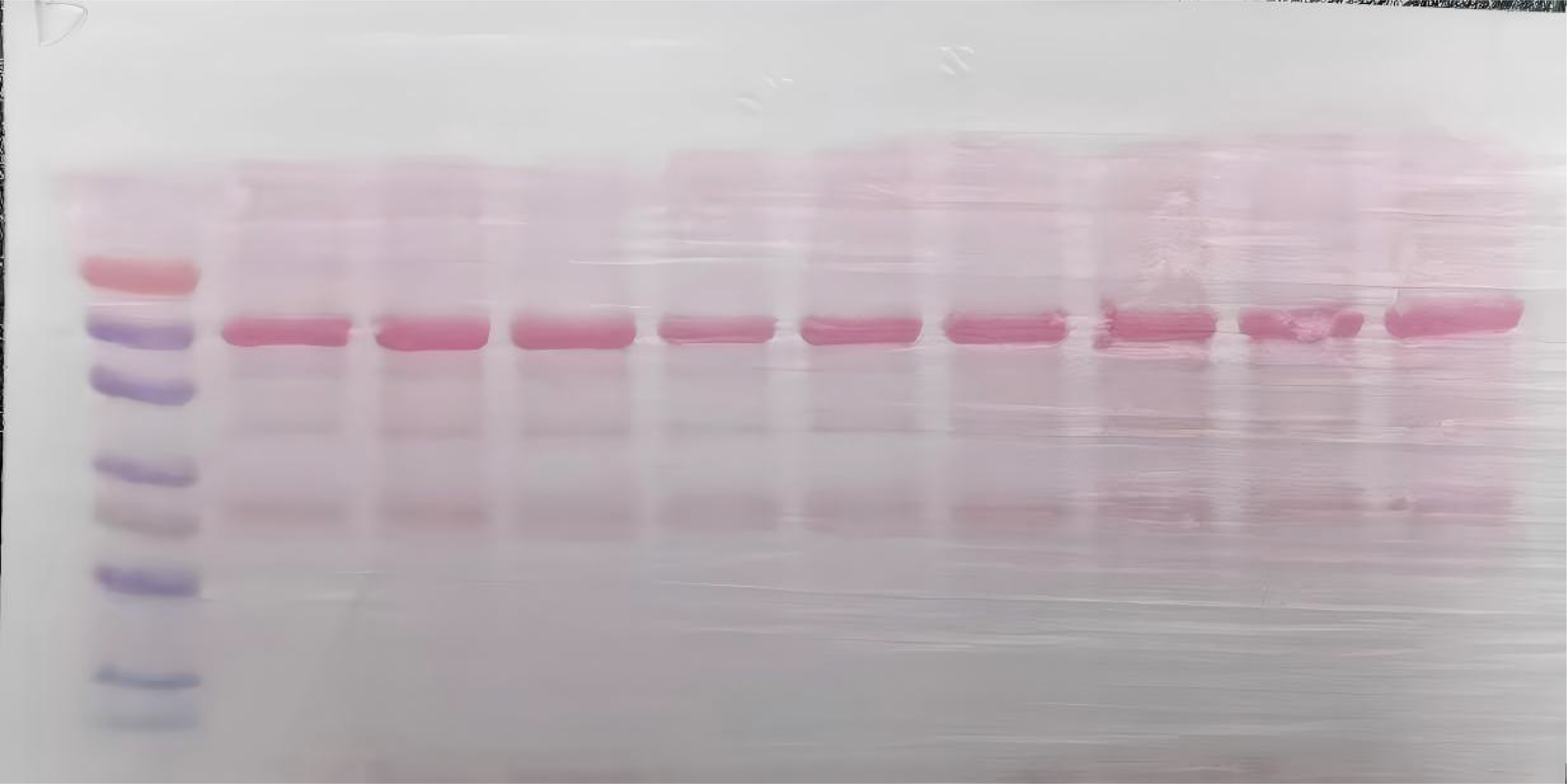

Supplement: Figure 2—source data 1. [file elife-88132-fig2-data1.zip › Figure 2-source data 1/Fig2C/Figure2-C-Ponceau S-RBCL.tif]

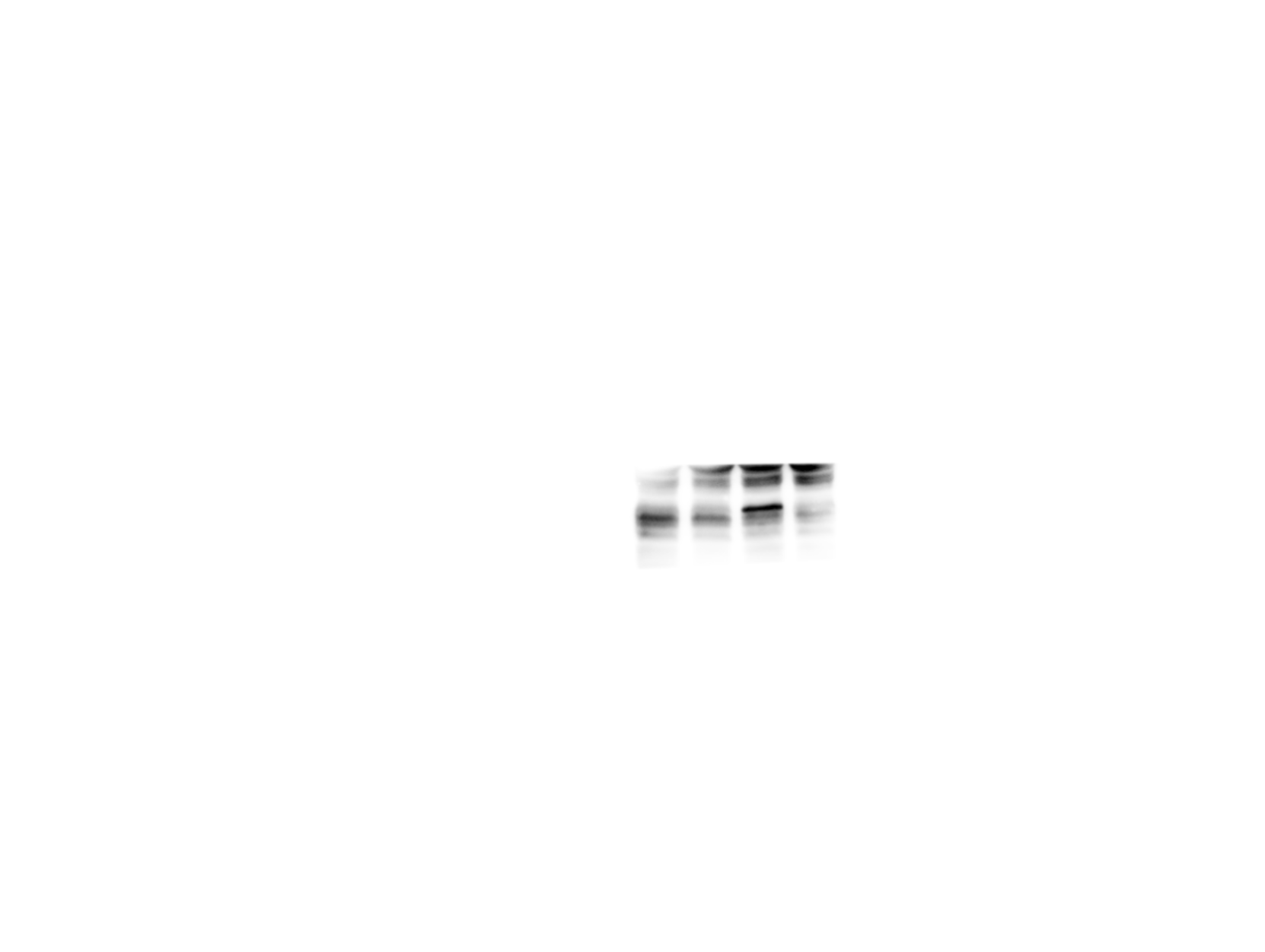

Supplement: Figure 2—source data 1. [file elife-88132-fig2-data1.zip › Figure 2-source data 1/Fig2I/Figure2-I-Blot1-anti-NP.tif]

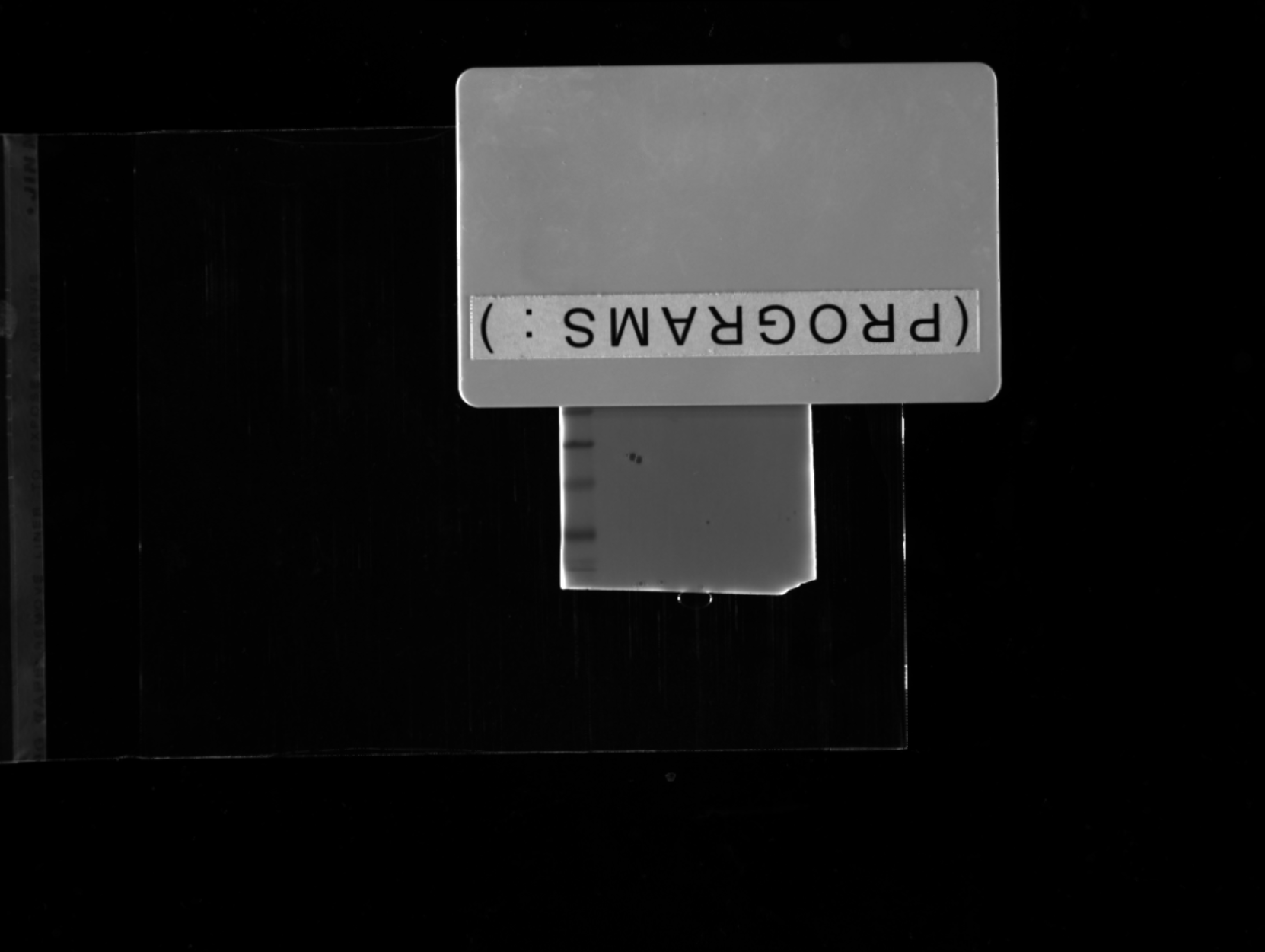

Supplement: Figure 2—source data 1. [file elife-88132-fig2-data1.zip › Figure 2-source data 1/Fig2I/Figure2-I-Blot1-marker.tif]

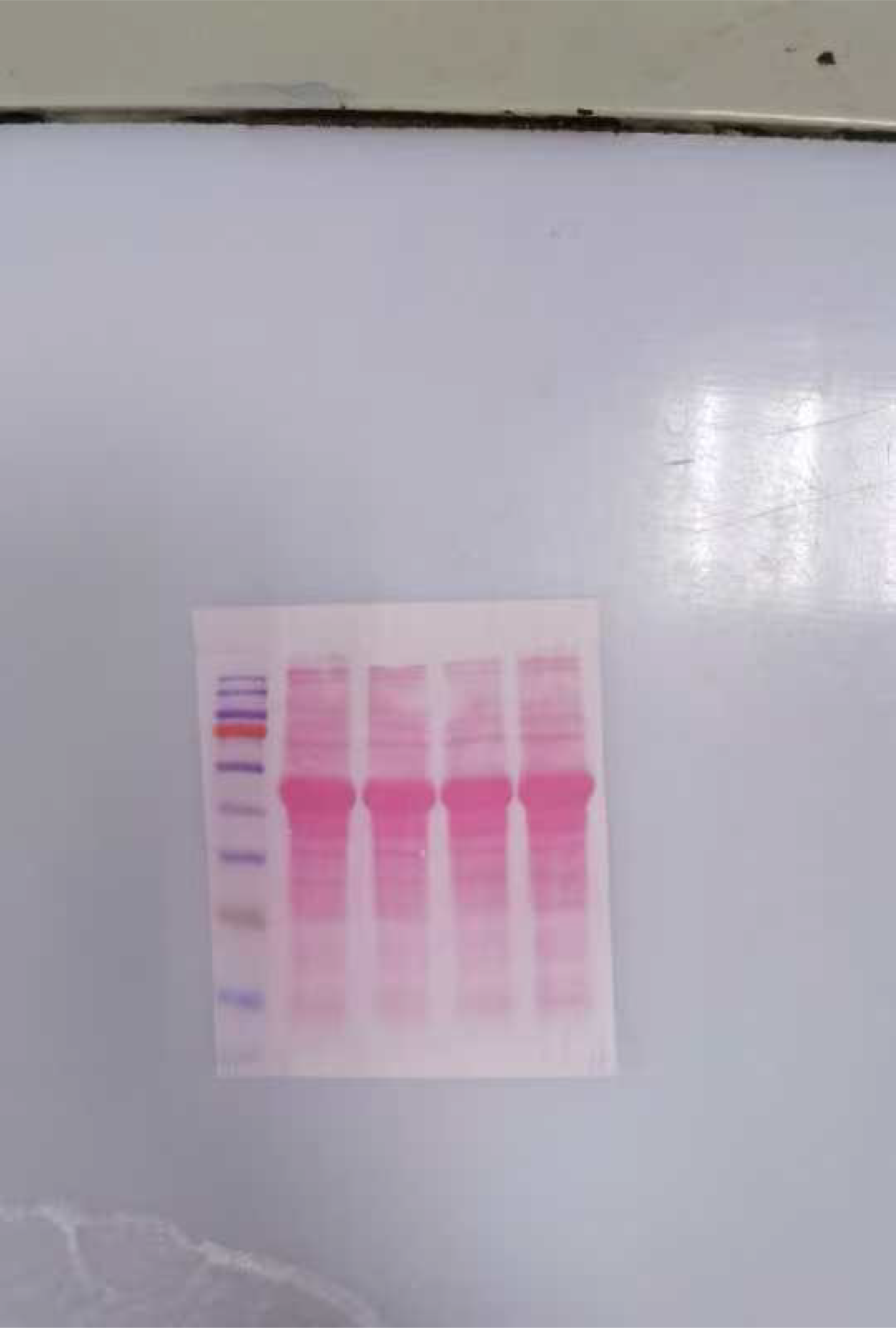

Supplement: Figure 2—source data 1. [file elife-88132-fig2-data1.zip › Figure 2-source data 1/Fig2I/Figure2-I-Ponceau S-RBCL.tif]

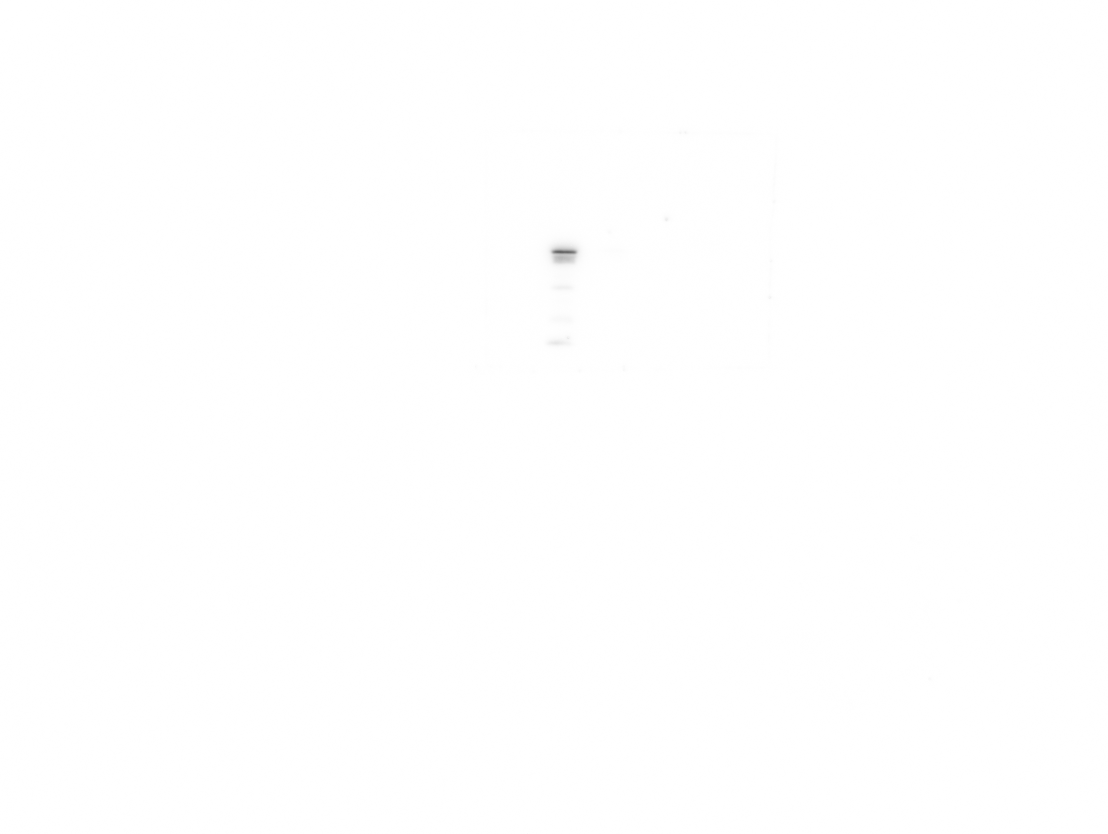

Supplement: Figure 3—source data 1. [file elife-88132-fig3-data1.zip › Figure 3-source data 1/Figure3-C-Blot1-anti-His.tif]

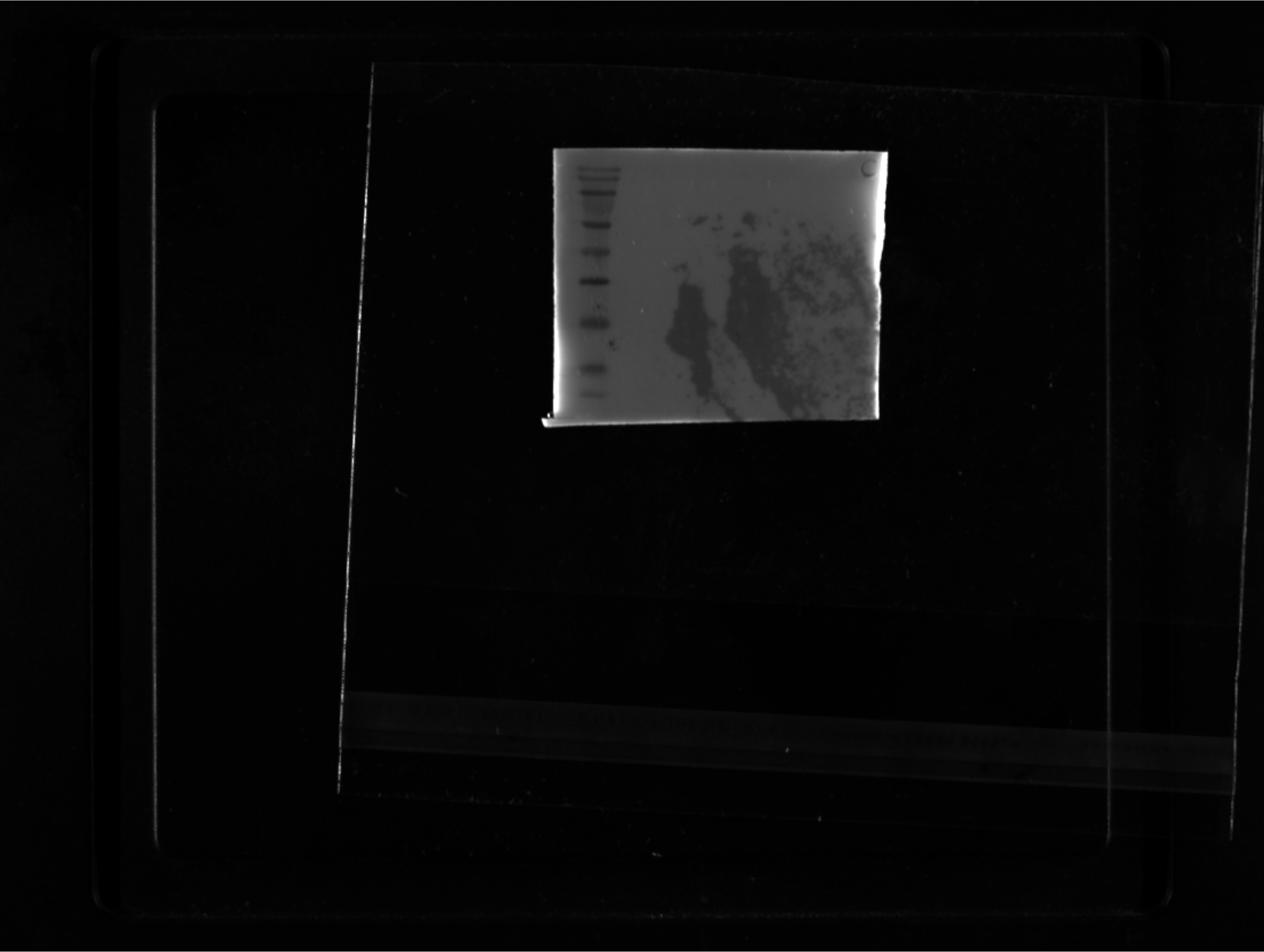

Supplement: Figure 3—source data 1. [file elife-88132-fig3-data1.zip › Figure 3-source data 1/Figure3-C-Blot1-marker.tif]

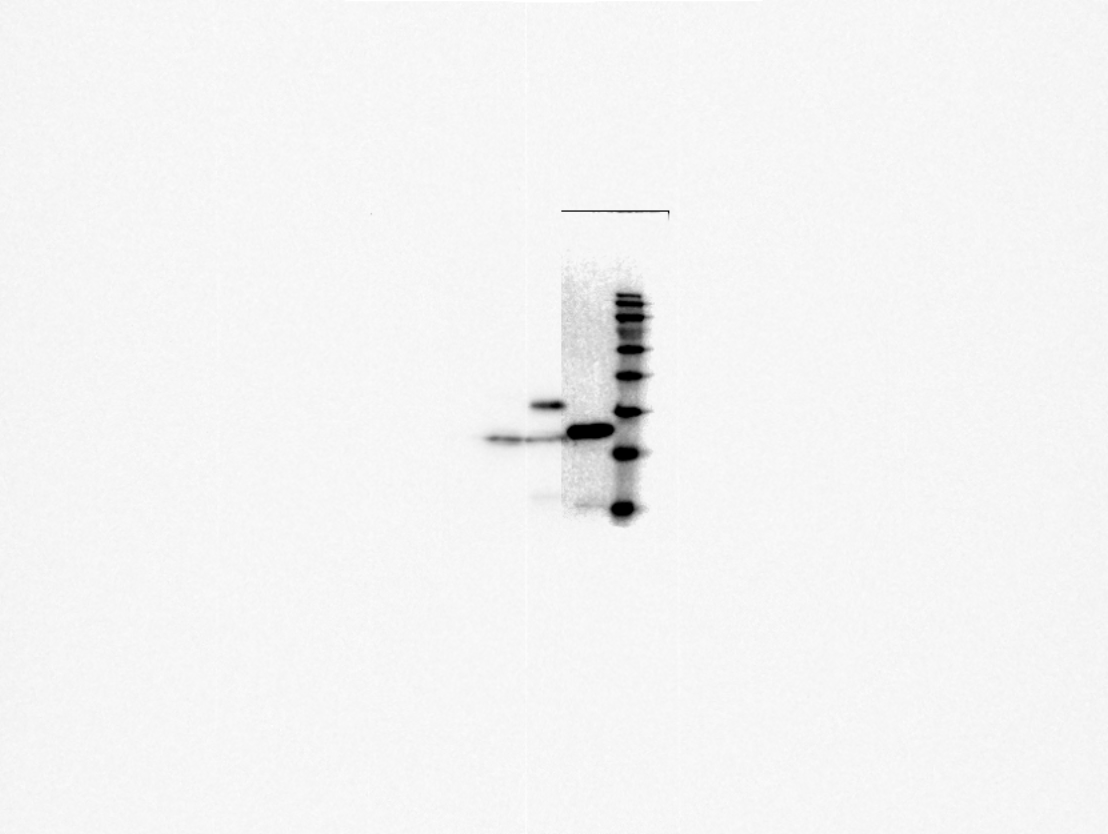

Supplement: Figure 3—source data 1. [file elife-88132-fig3-data1.zip › Figure 3-source data 1/Figure3-C-Blot2-anti-His.tif]

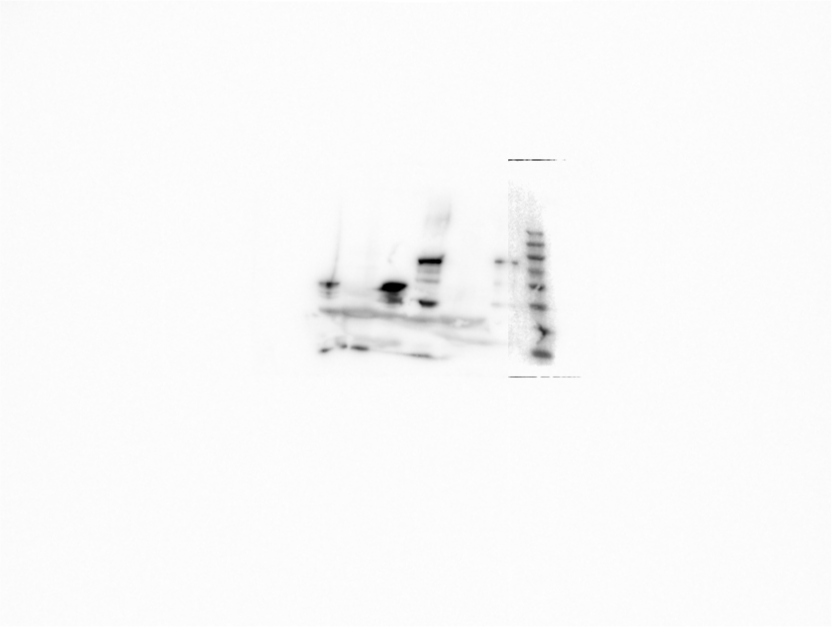

Supplement: Figure 3—source data 1. [file elife-88132-fig3-data1.zip › Figure 3-source data 1/Figure3-C-Blot3-anti-MBP.tif]

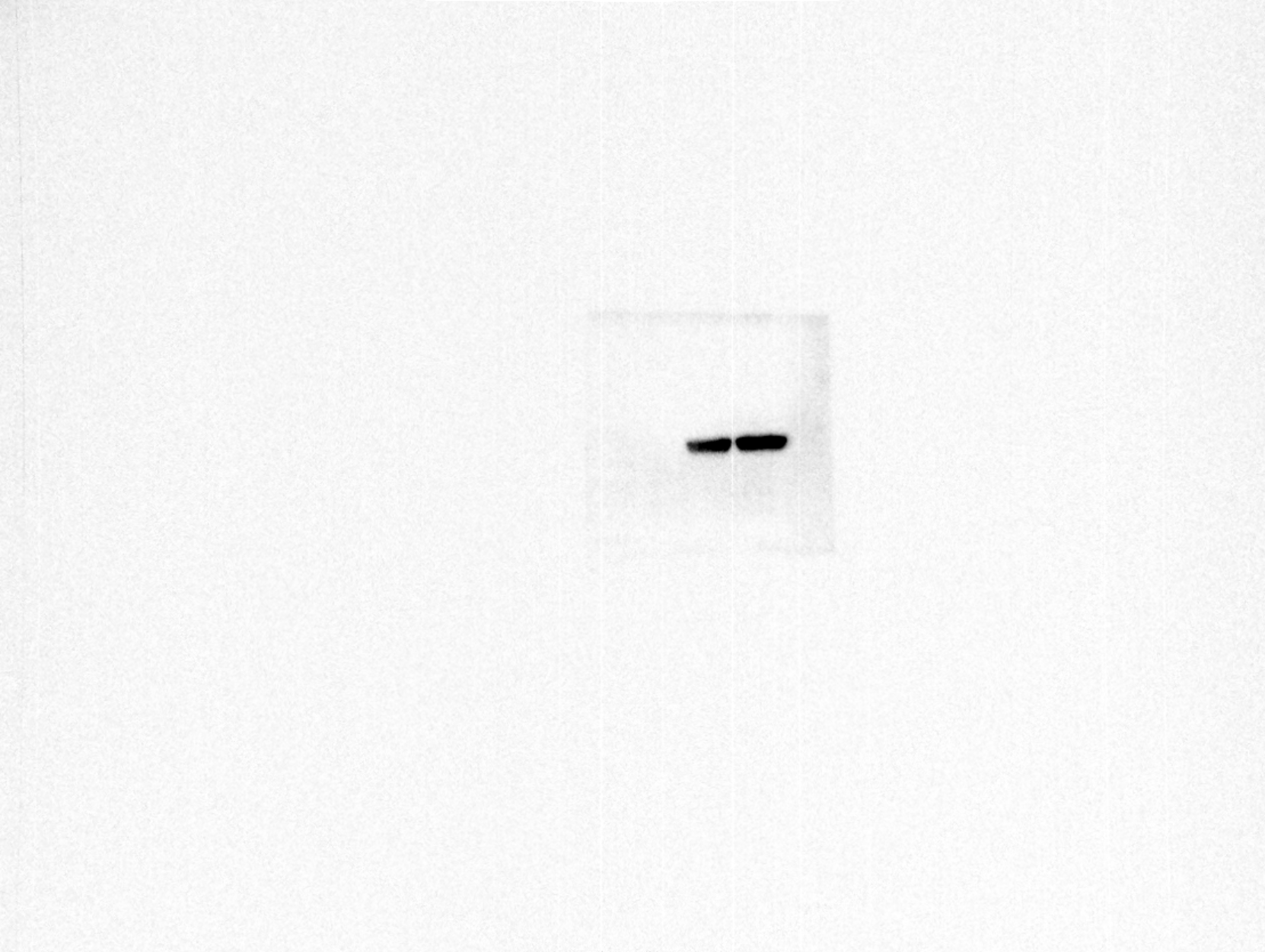

Supplement: Figure 3—figure supplement 2—source data 1. [file elife-88132-fig3-figsupp2-data1.zip › Figure 3-figure supplement 2-source data 1/Figure3-supplment2-anti-His.tif]

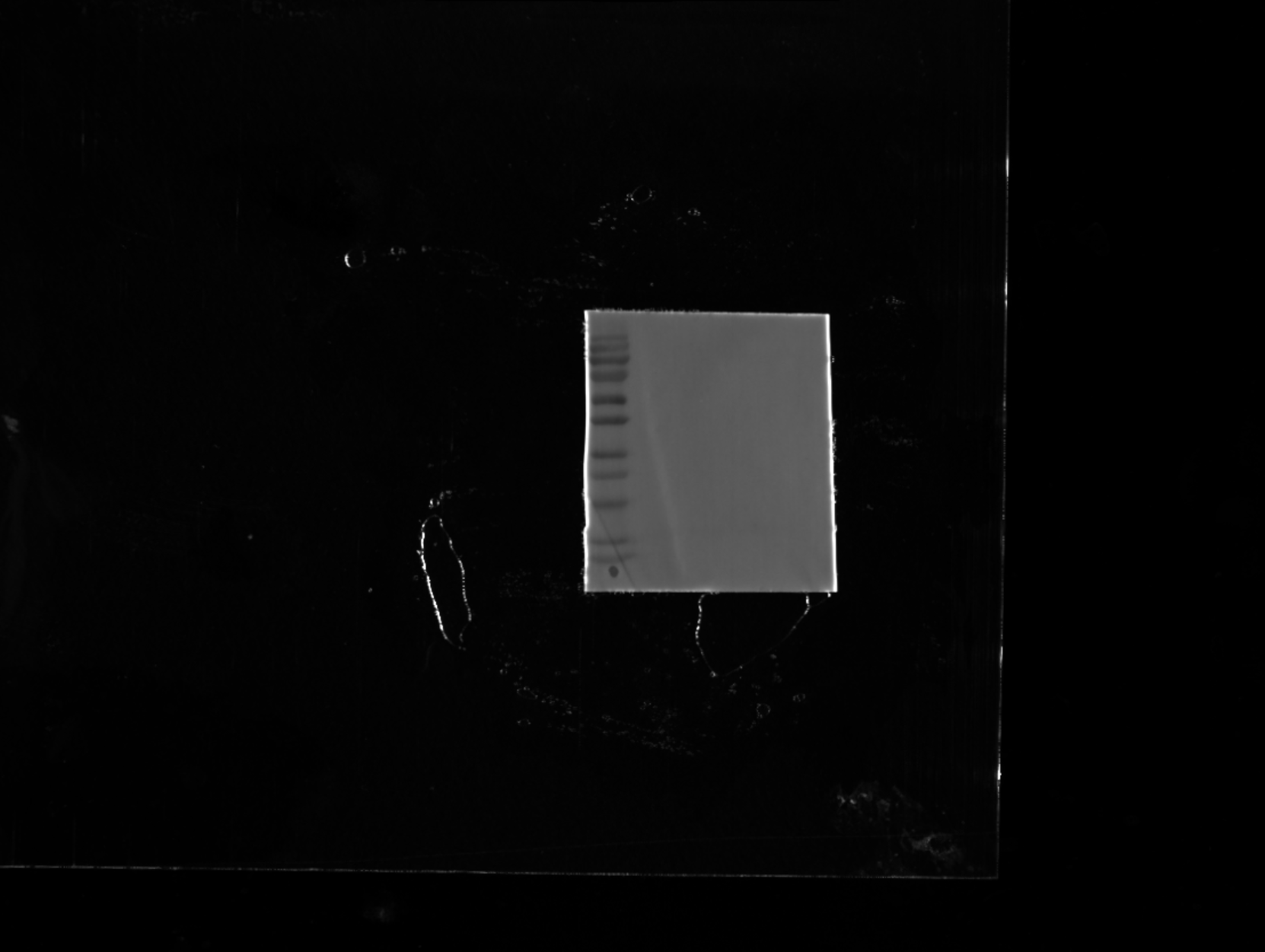

Supplement: Figure 3—figure supplement 2—source data 1. [file elife-88132-fig3-figsupp2-data1.zip › Figure 3-figure supplement 2-source data 1/Figure3-supplment2-marker.tif]

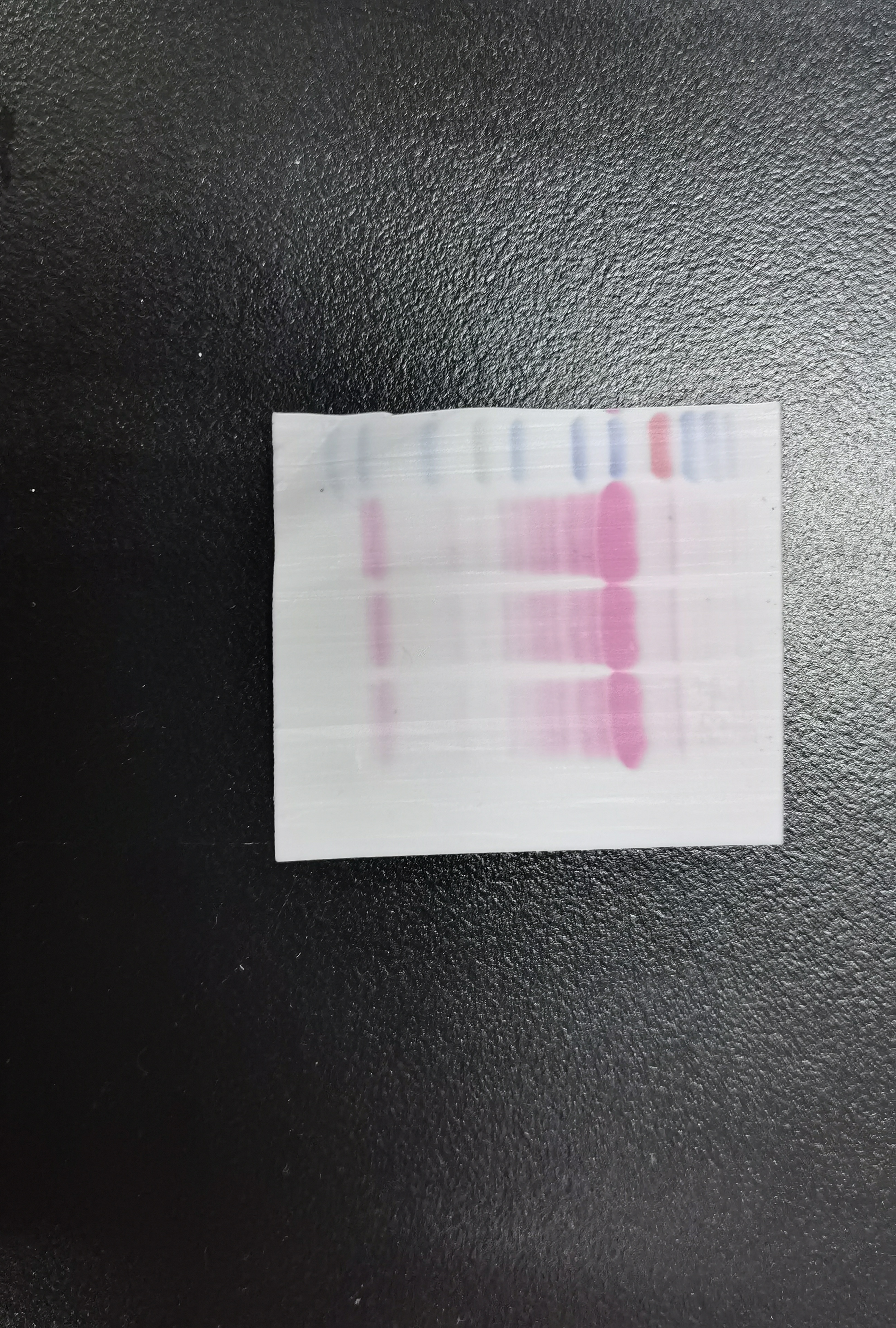

Supplement: Figure 3—figure supplement 2—source data 1. [file elife-88132-fig3-figsupp2-data1.zip › Figure 3-figure supplement 2-source data 1/Figure3-supplment2-Ponceau S-RBCL.jpg]

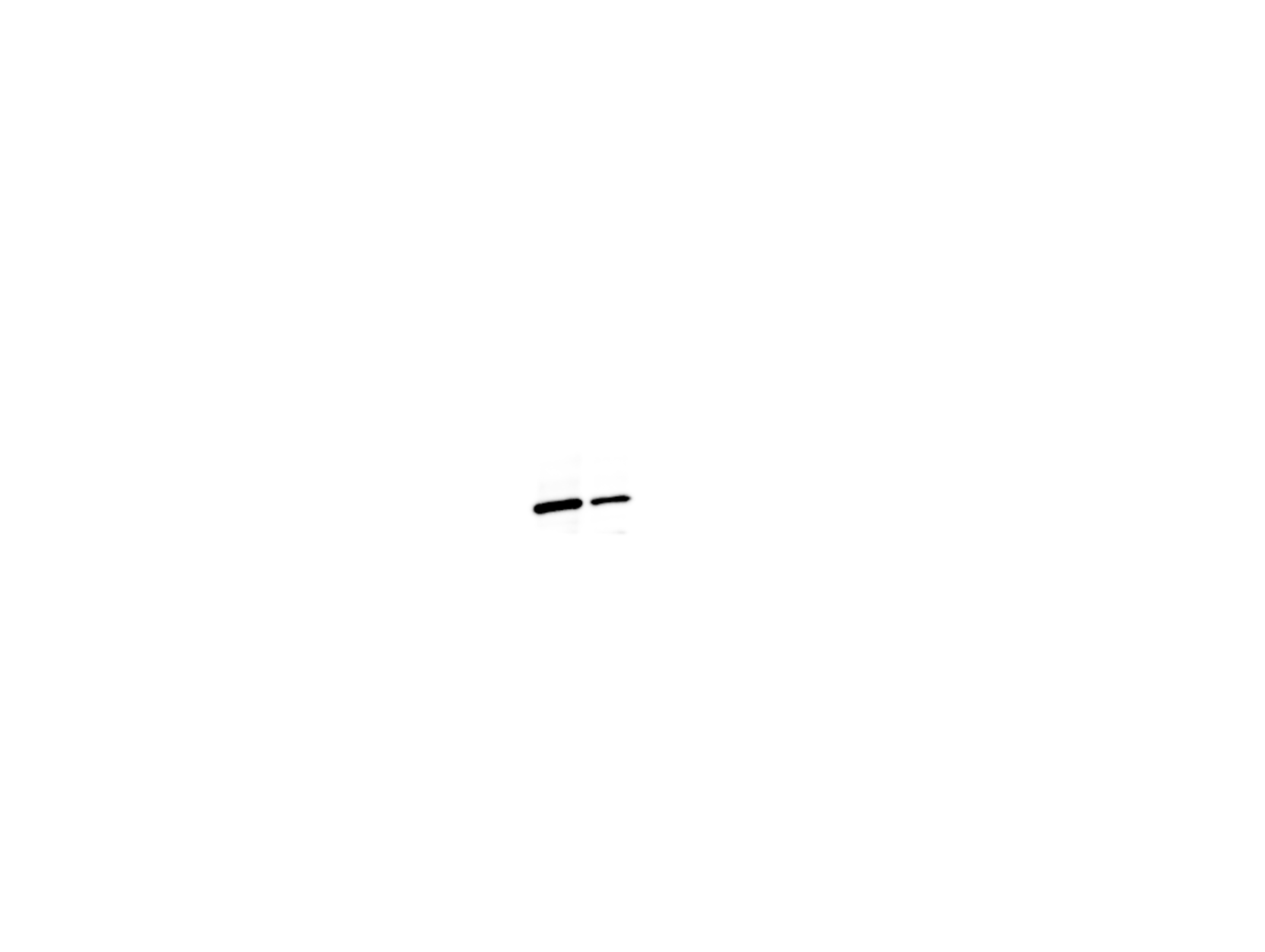

Supplement: Figure 4—source data 1. [file elife-88132-fig4-data1.zip › Figure 4-source data 1/Figure4-A-Blot1-anti-His.tif]

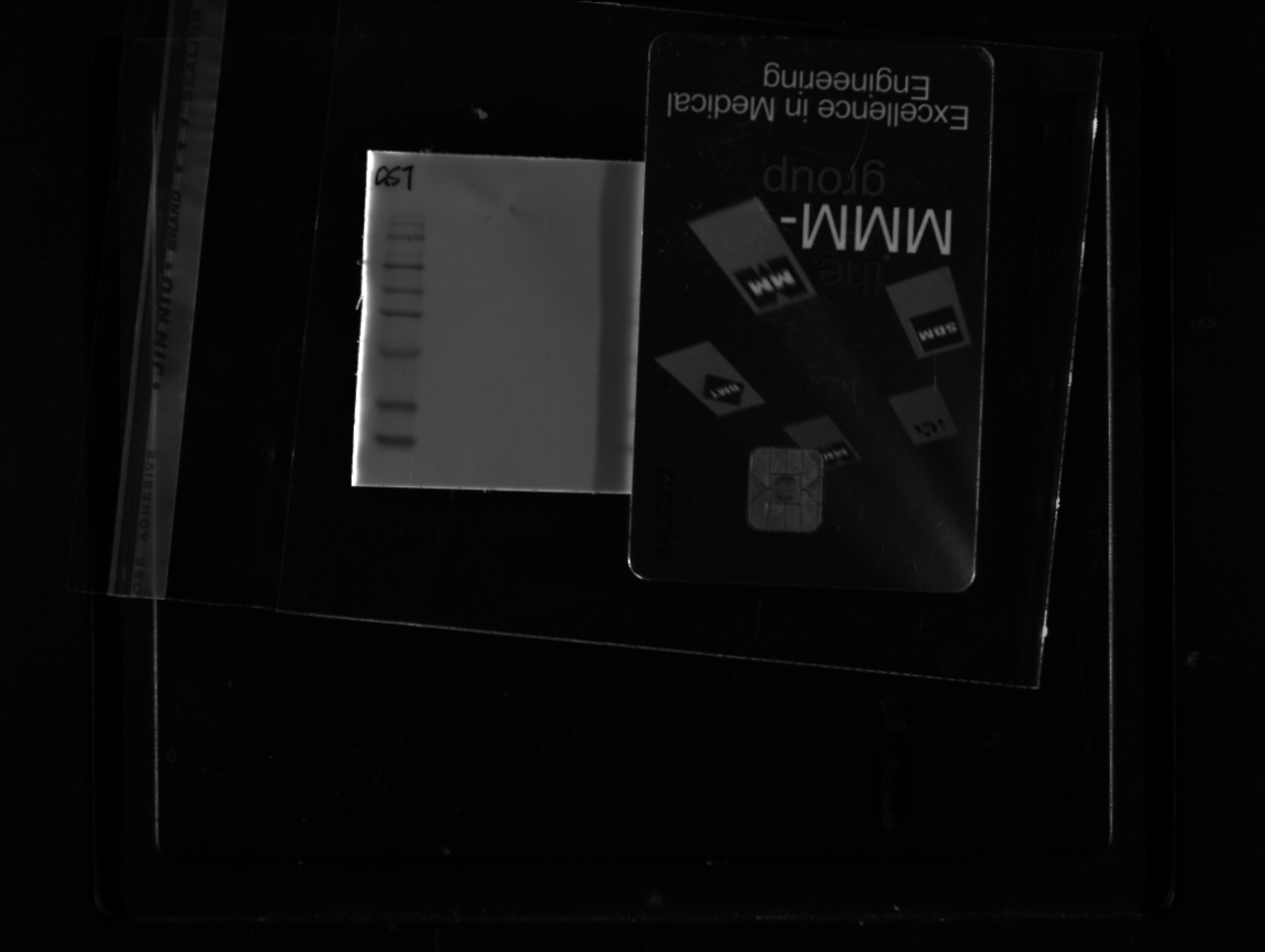

Supplement: Figure 4—source data 1. [file elife-88132-fig4-data1.zip › Figure 4-source data 1/Figure4-A-Blot1-marker.tif]

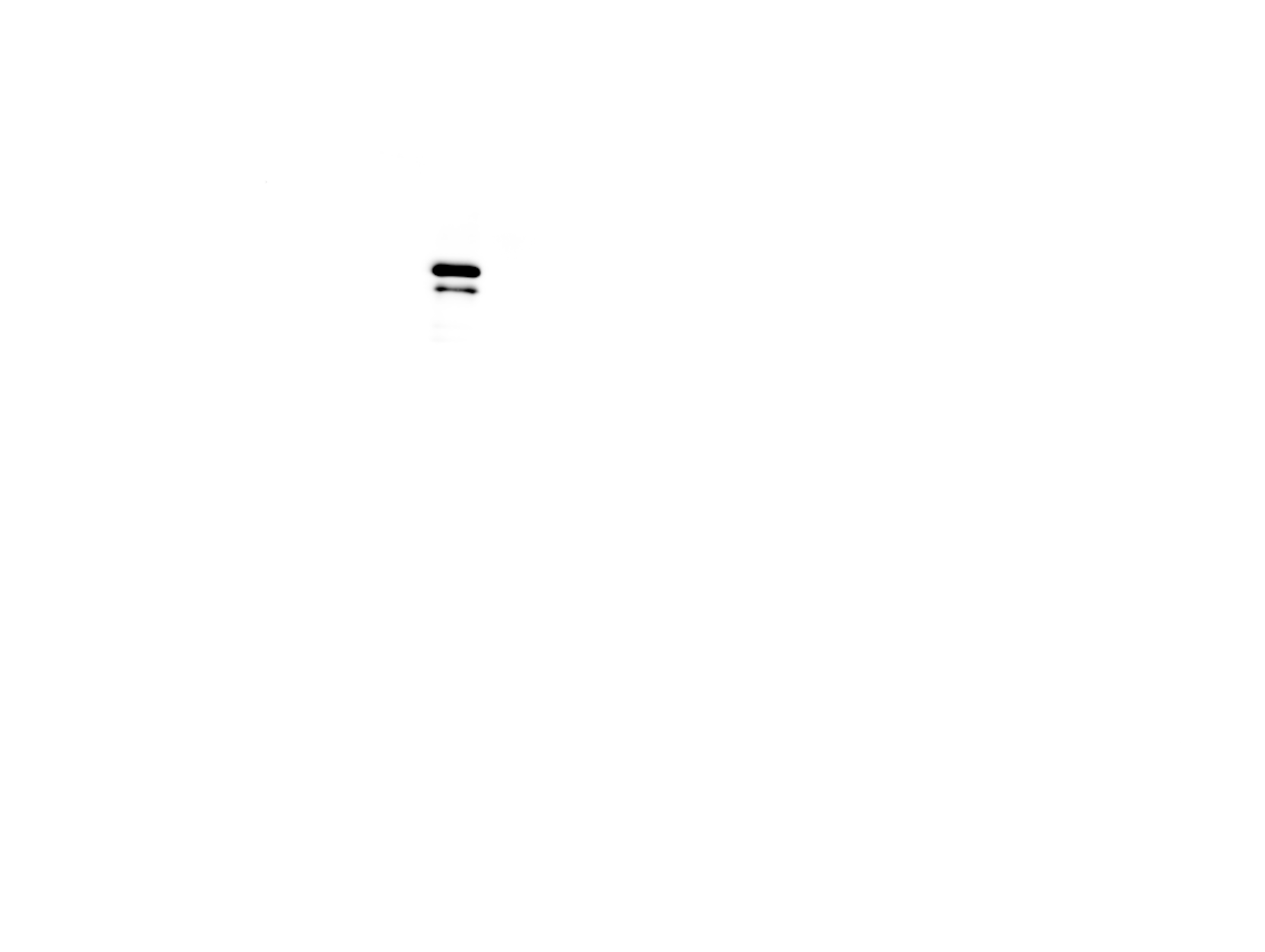

Supplement: Figure 4—source data 1. [file elife-88132-fig4-data1.zip › Figure 4-source data 1/Figure4-A-Blot2-anti-GST.tif]

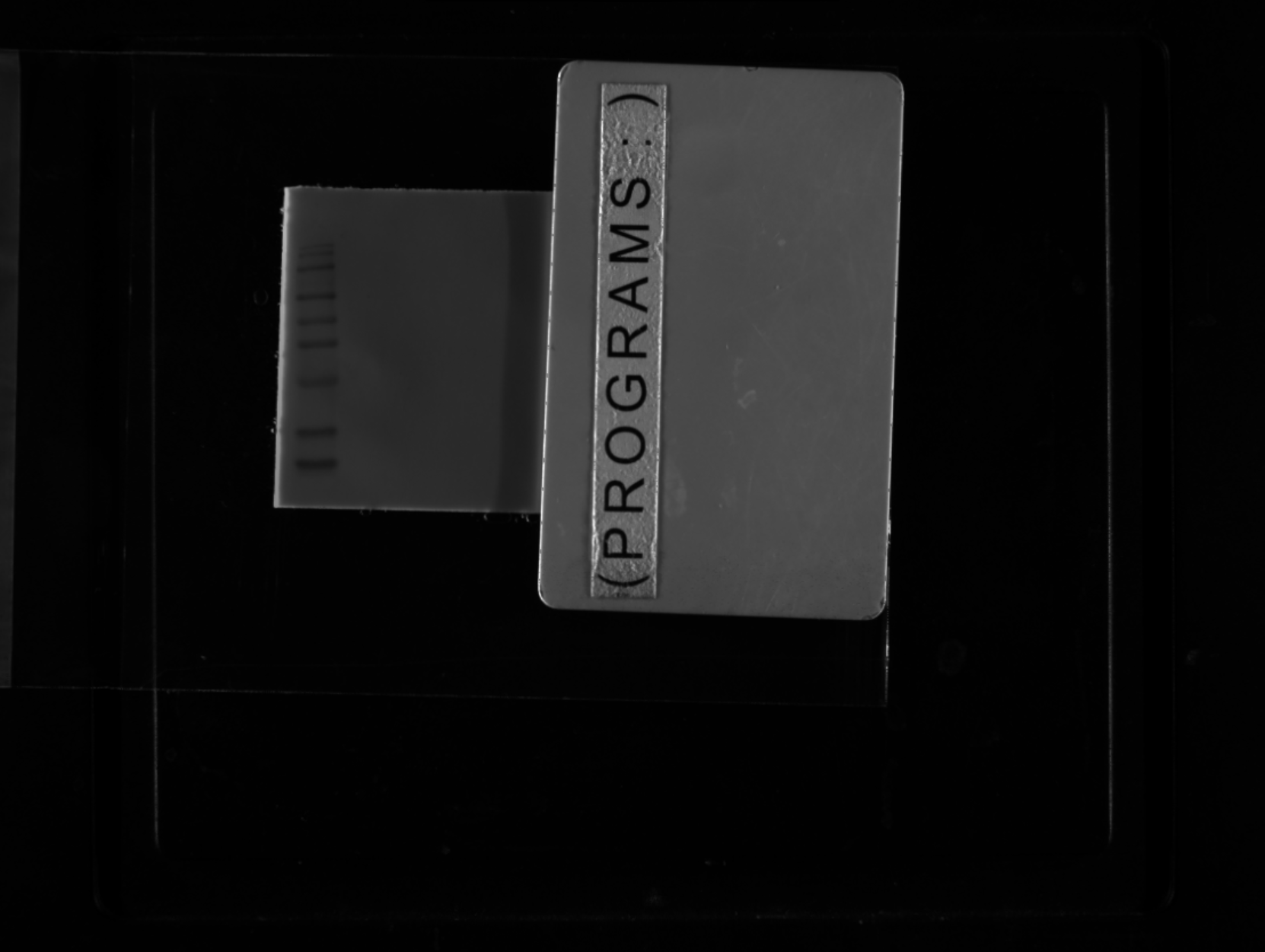

Supplement: Figure 4—source data 1. [file elife-88132-fig4-data1.zip › Figure 4-source data 1/Figure4-A-Blot2-marker.tif]

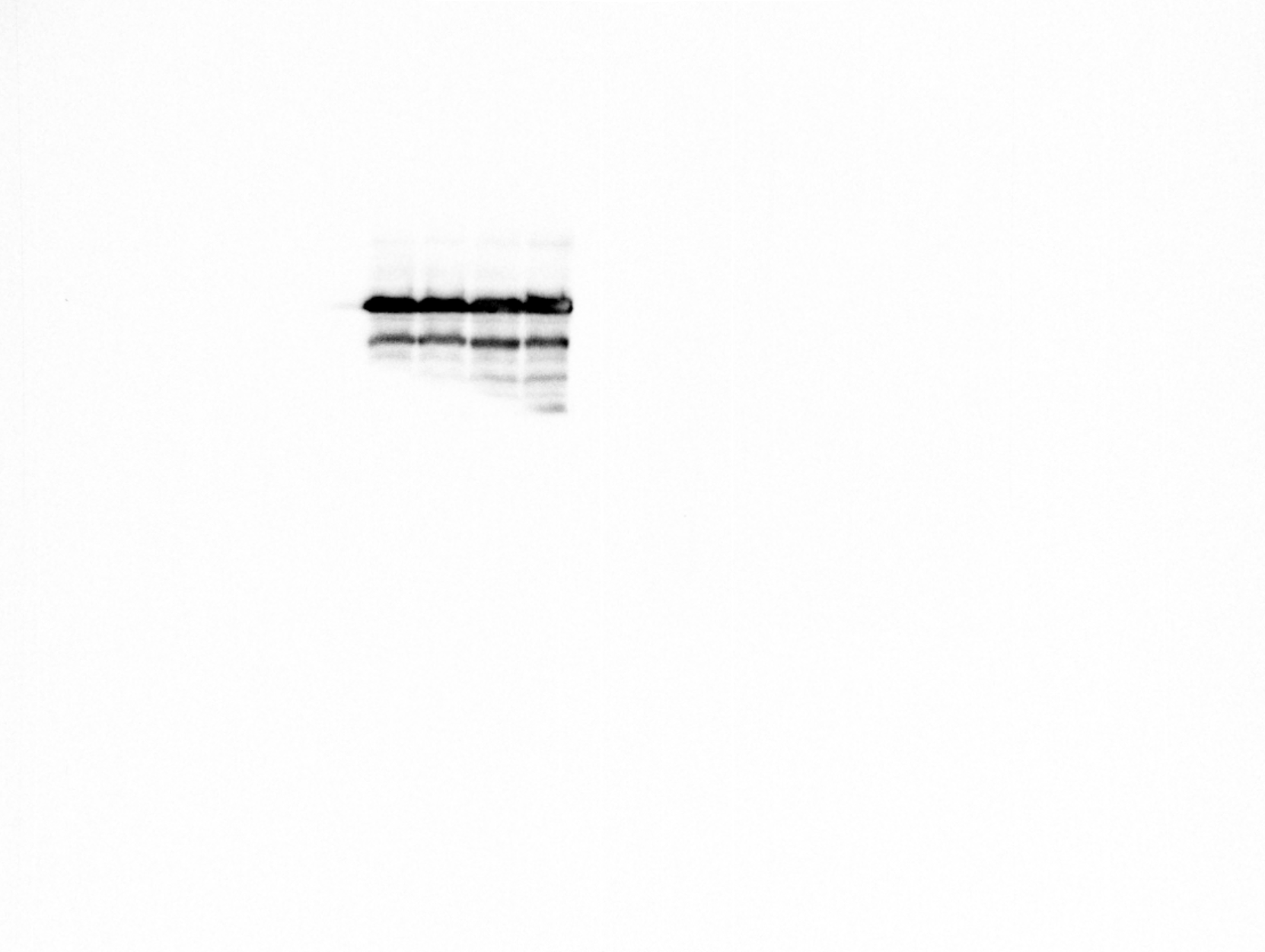

Supplement: Figure 4—source data 1. [file elife-88132-fig4-data1.zip › Figure 4-source data 1/Figure4-A-Blot3-anti-His.tif]

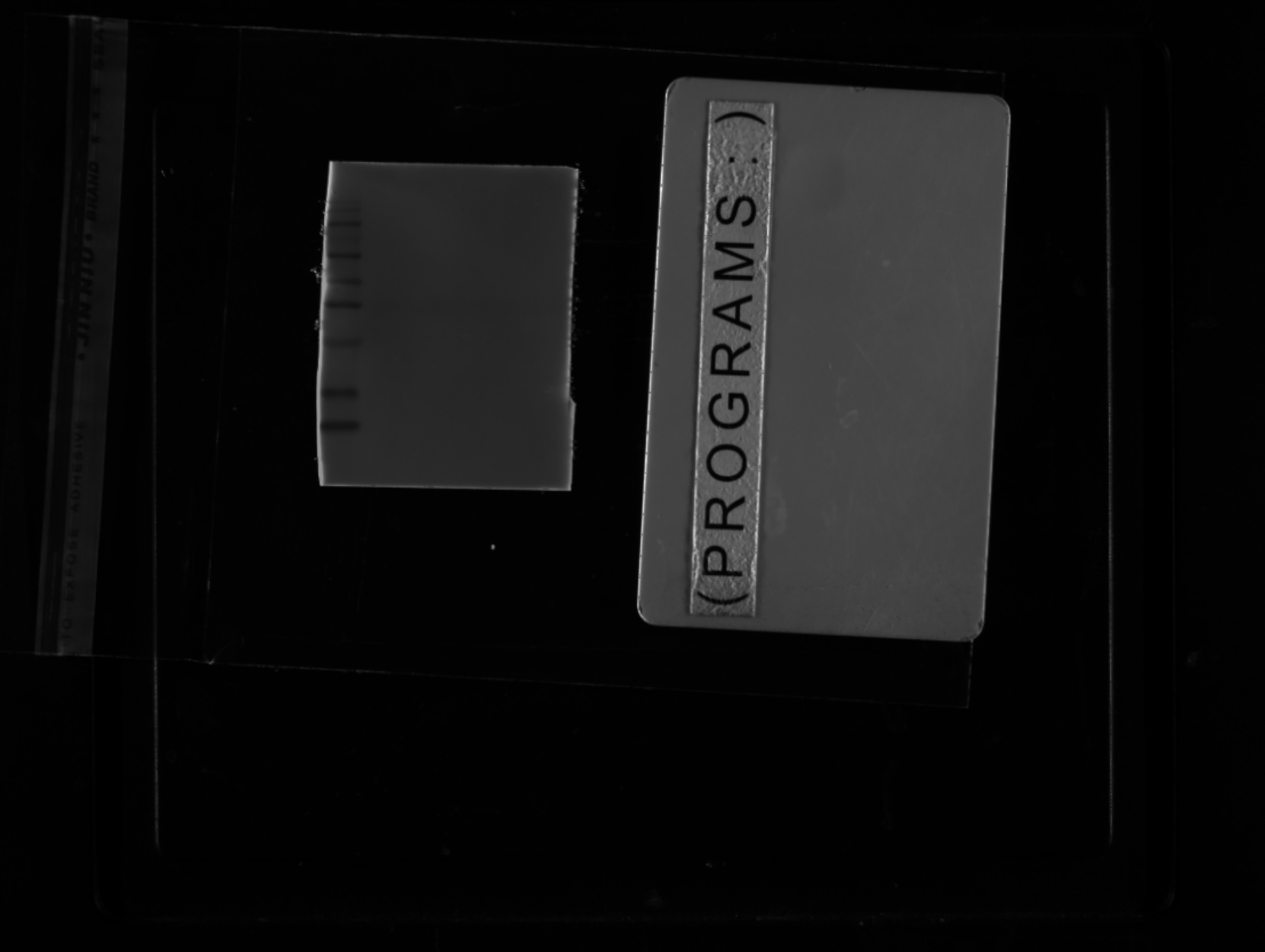

Supplement: Figure 4—source data 1. [file elife-88132-fig4-data1.zip › Figure 4-source data 1/Figure4-A-Blot3-marker.tif]

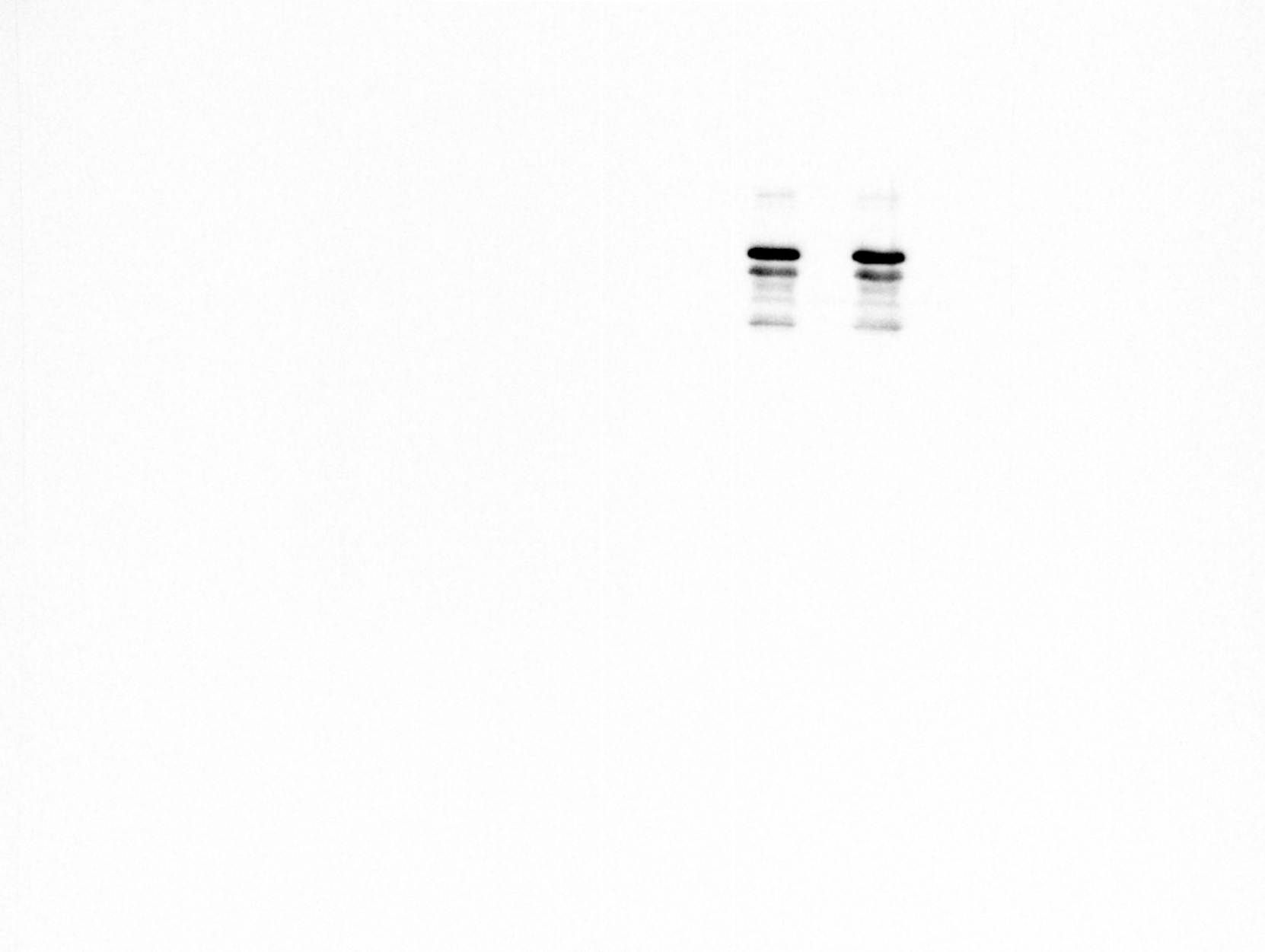

Supplement: Figure 4—source data 1. [file elife-88132-fig4-data1.zip › Figure 4-source data 1/Figure4-A-Blot4-anti-GST.tif]

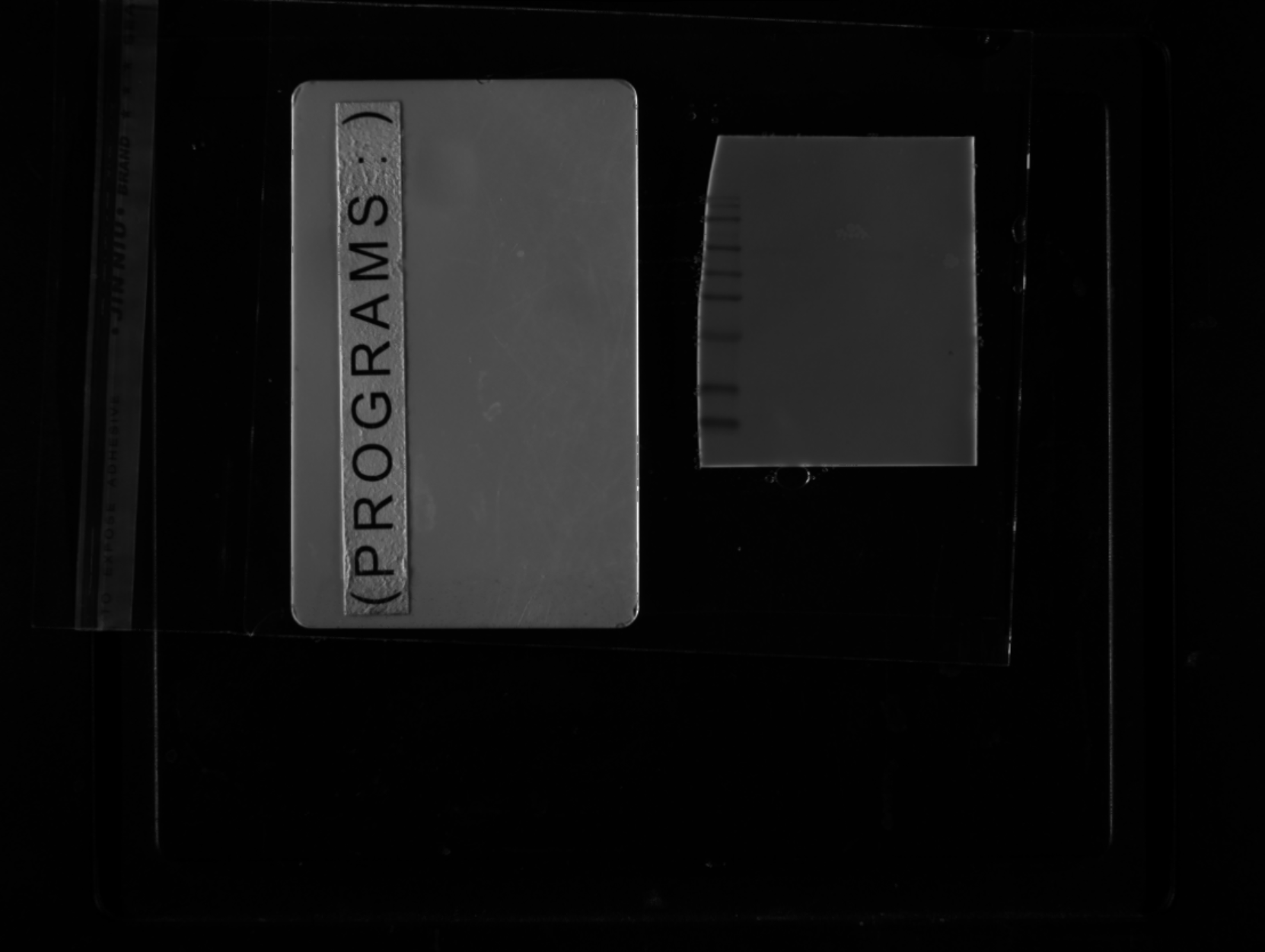

Supplement: Figure 4—source data 1. [file elife-88132-fig4-data1.zip › Figure 4-source data 1/Figure4-A-Blot4-marker.tif]

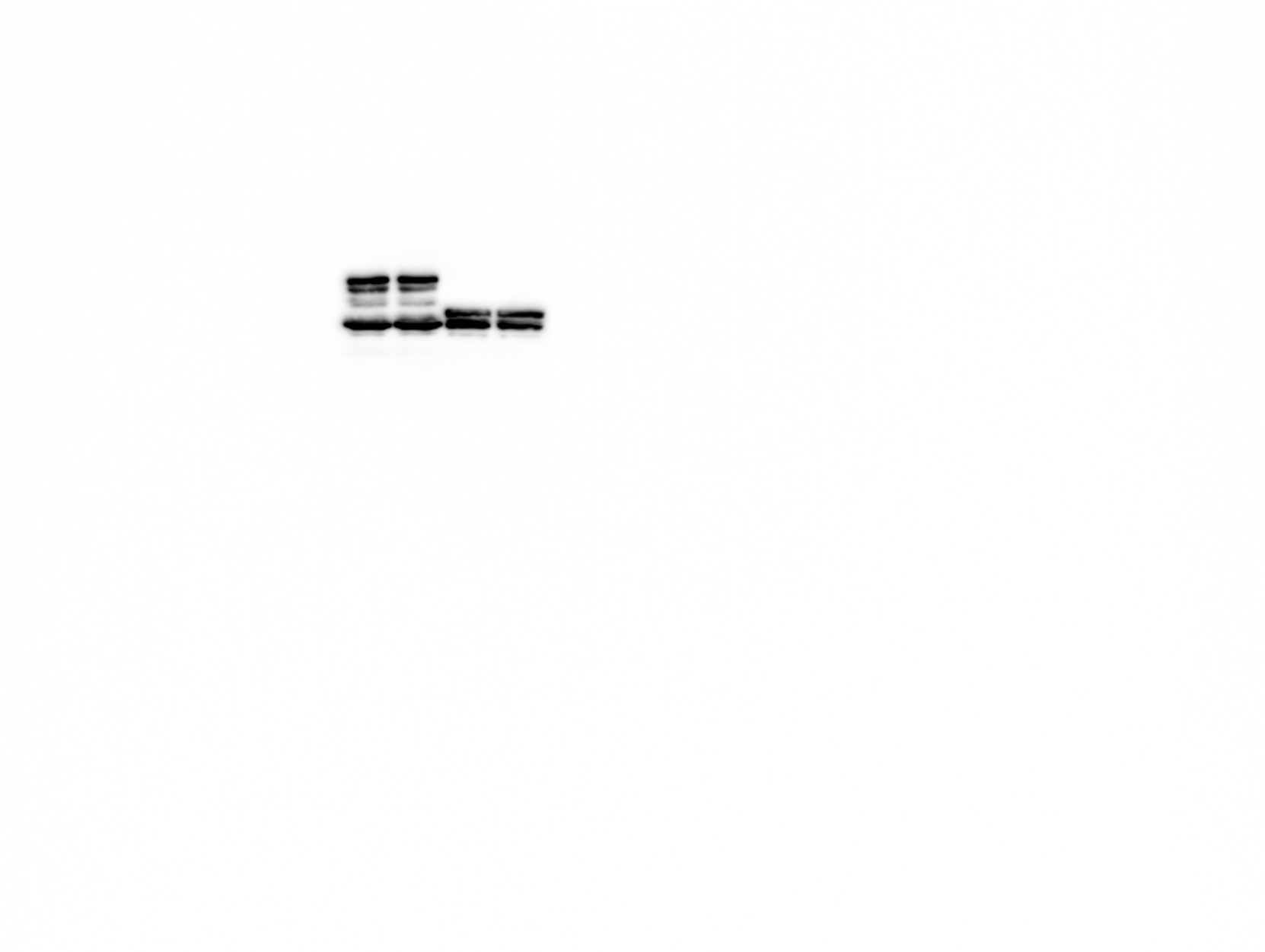

Supplement: Figure 4—source data 1. [file elife-88132-fig4-data1.zip › Figure 4-source data 1/Figure4-A-Blot5-anti-MBP.tif]

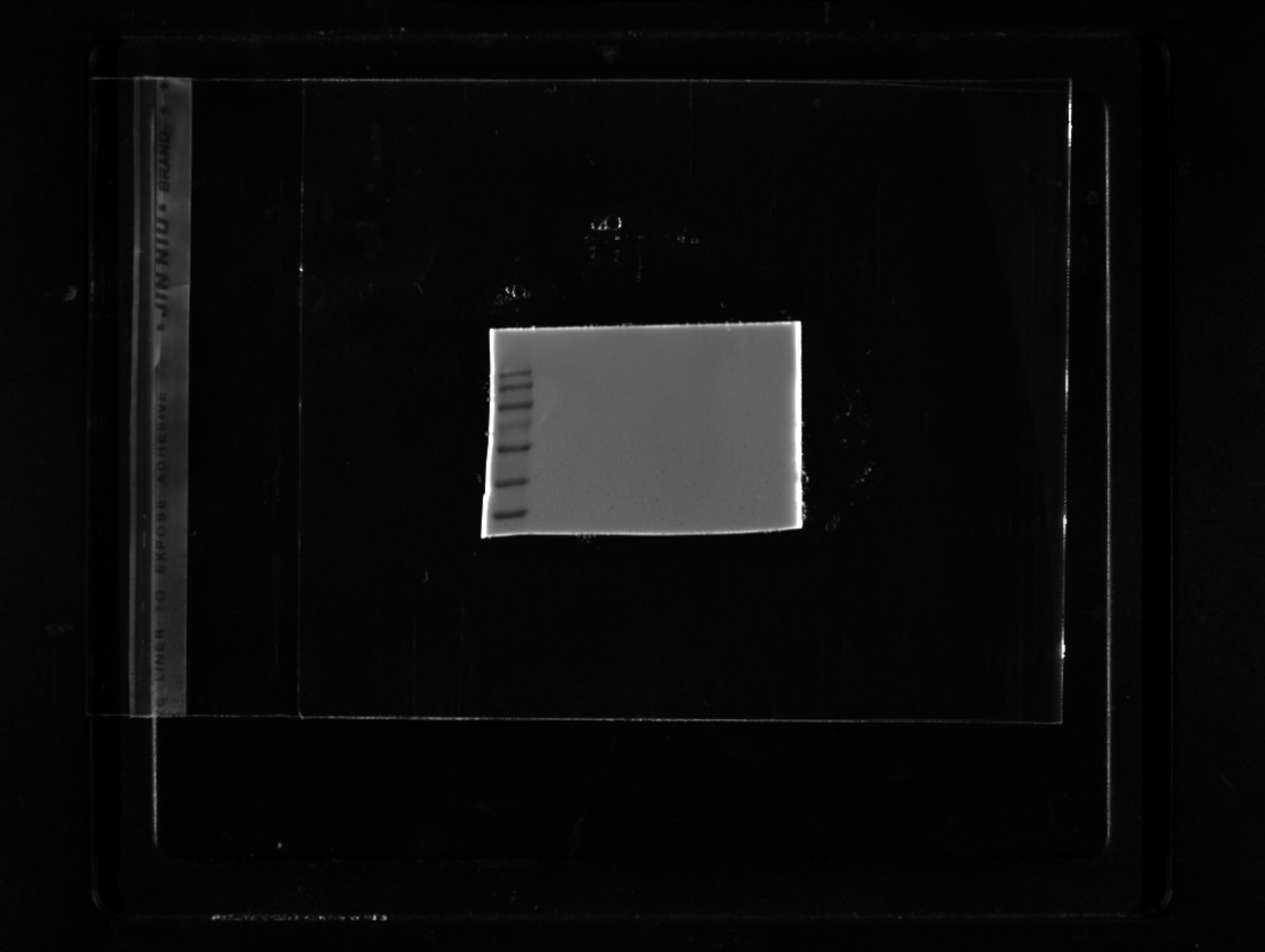

Supplement: Figure 4—source data 1. [file elife-88132-fig4-data1.zip › Figure 4-source data 1/Figure4-A-Blot5-marker.tif]
