# Supplementary material for: Rice stripe virus utilizes a Laodelphax striatellus salivary carbonic anhydrase to facilitate plant infection by direct molecular interaction
Source: eLife. 2026 Jan 6;12:RP88132. doi: 10.7554/eLife.88132 (PMC12774414; doi:10.7554/eLife.88132)
Supplement: Figure 1—source data 2. [file elife-88132-fig1-data2.zip › Figure 1-source data 2/Figure1-I-Source data.pdf]

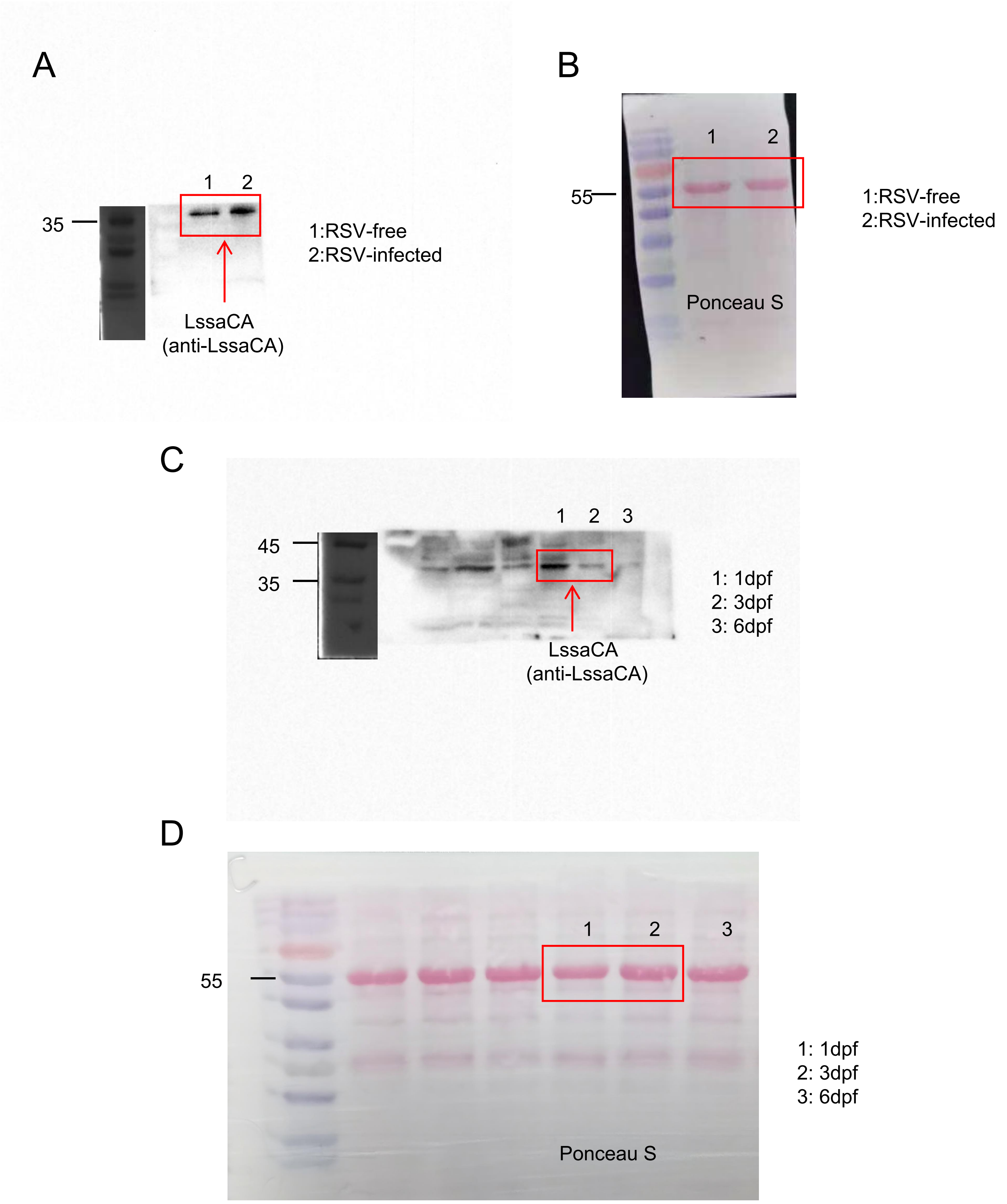

Figure1-I-Source data 2. Original membranes corresponding to Figure 1, panel I. Rainbow molecular weight markers were employed. Panels A & B: Expression analysis in control and knockdown conditions. (A) Western blot of target protein. (B) Corresponding Ponceau S staining of the same membrane, showing RBCL as the loading control. Lanes: 1, dsGFP control; 2, dsLssaCA. Panels C & D: Protein levels at different time points post-infection. (C) Western blot of target protein. (D) Corresponding Ponceau S staining (RBCL loading control). Lanes: 1, 1 dpi; 2, 3 dpi; 3, 6 dpi (data not shown). The antibodies used for detection are indicated on the figure.
