# Supplementary material for: Rice stripe virus utilizes a Laodelphax striatellus salivary carbonic anhydrase to facilitate plant infection by direct molecular interaction
Source: eLife. 2026 Jan 6;12:RP88132. doi: 10.7554/eLife.88132 (PMC12774414; doi:10.7554/eLife.88132)
Supplement: Figure 1—figure supplement 3—source data 2. [file elife-88132-fig1-figsupp3-data2.zip › Figure 1-figure supplement 3-source data 2/Figure1-supplement3-Source data.pdf]

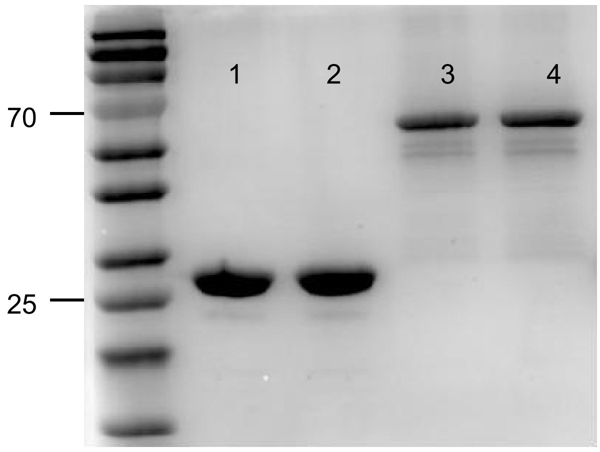

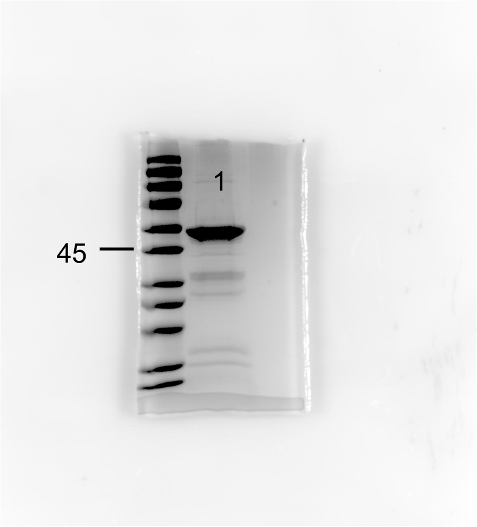

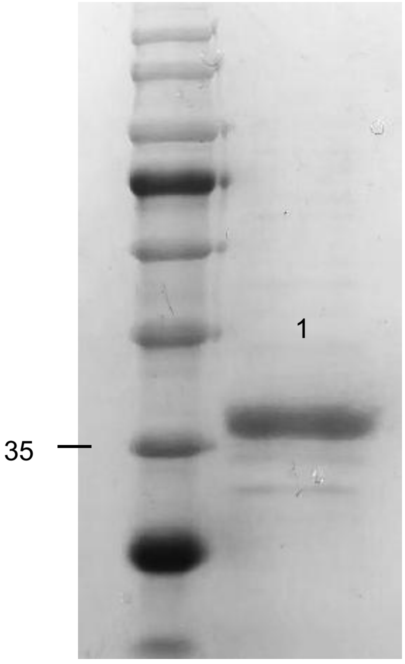

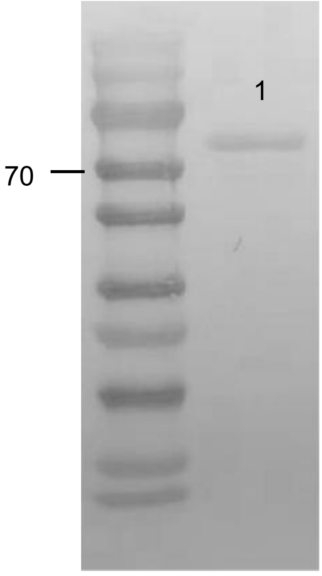

Figure1-supplement3-Source data 2. Original membranes corresponding to Figure 1, supplement3. Rainbow molecular weight markers were employed. Shown is a Coomassie brilliant blue-stained SDS-PAGE gel of purified recombinant proteins used in the MicroScale Thermophoresis (MST) assay.
