# Supplementary material for: Rice stripe virus utilizes a Laodelphax striatellus salivary carbonic anhydrase to facilitate plant infection by direct molecular interaction
Source: eLife. 2026 Jan 6;12:RP88132. doi: 10.7554/eLife.88132 (PMC12774414; doi:10.7554/eLife.88132)
Supplement: Figure 2—source data 2. [file elife-88132-fig2-data2.zip › Figure 2-source data 2/Figure2-C-Source data.pdf]

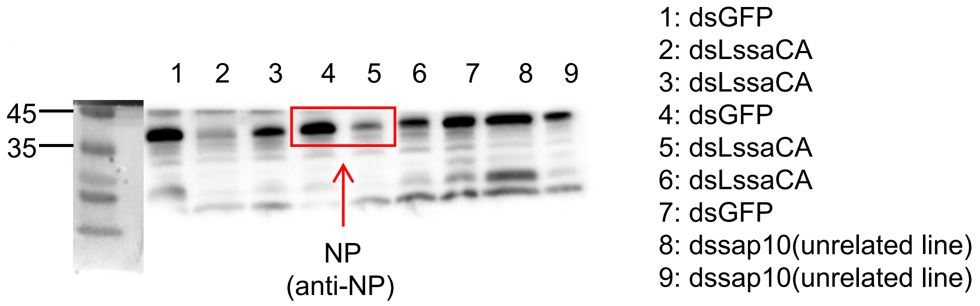

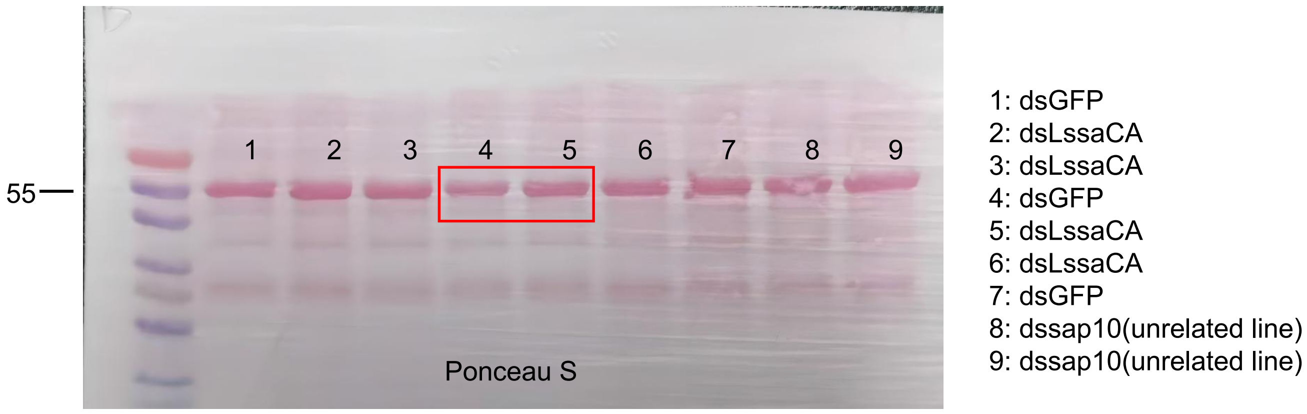

Figure2-C-Source data 2. Original membranes corresponding to Figure 2, panel C. Rainbow molecular weight markers were employed. Western blot of the target protein (top) and Ponceau S staining (bottom). RBCL as loading control. Only relevant lanes are shown: lane 4 (dsGFP) and lane 5 (dsLssaCA). Remaining lanes are omitted/unrelated. The antibodies used for detection are indicated on the figure.
