# Supplementary material for: Rice stripe virus utilizes a Laodelphax striatellus salivary carbonic anhydrase to facilitate plant infection by direct molecular interaction
Source: eLife. 2026 Jan 6;12:RP88132. doi: 10.7554/eLife.88132 (PMC12774414; doi:10.7554/eLife.88132)
Supplement: Figure 3—source data 2. [file elife-88132-fig3-data2.zip › Figure 3-source data 2/Figure3-C-Source data.pdf]

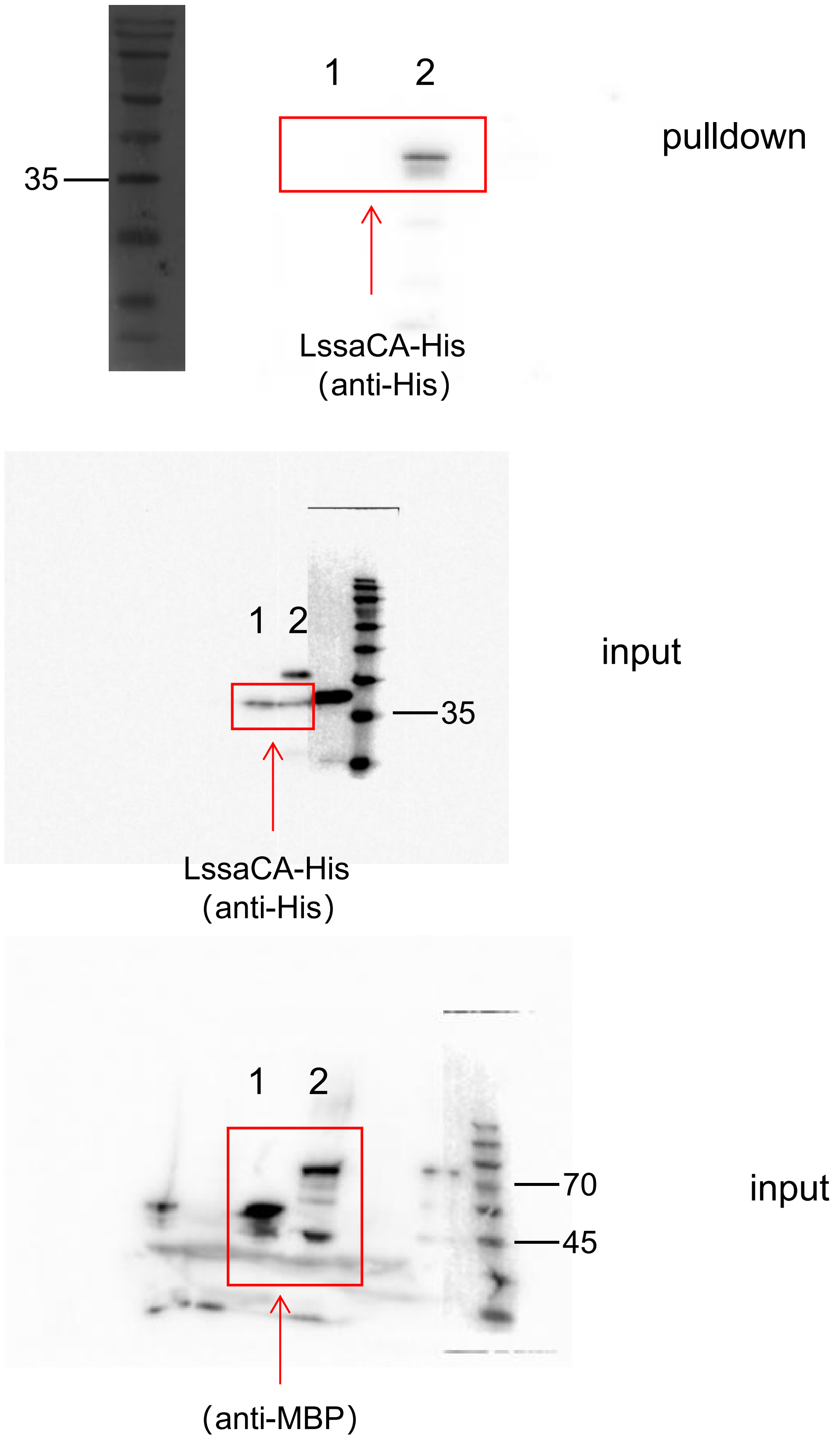

Figure3-C-Source data 2. Original membranes corresponding to Figure 3, panel C. Rainbow molecular weight markers were employed. Lane 1: MBP co-incubated with NP-His. Lane 2: MBP-OsTLP co-incubated with NP-His. The antibodies used for detection are indicated on the figure.
