# Supplementary material for: Rice stripe virus utilizes a Laodelphax striatellus salivary carbonic anhydrase to facilitate plant infection by direct molecular interaction
Source: eLife. 2026 Jan 6;12:RP88132. doi: 10.7554/eLife.88132 (PMC12774414; doi:10.7554/eLife.88132)
Supplement: Figure 3—figure supplement 2—source data 2. [file elife-88132-fig3-figsupp2-data2.zip › Figure 3-figure supplement 2-source data 2/Figure3-supplement2-Source data.pdf]

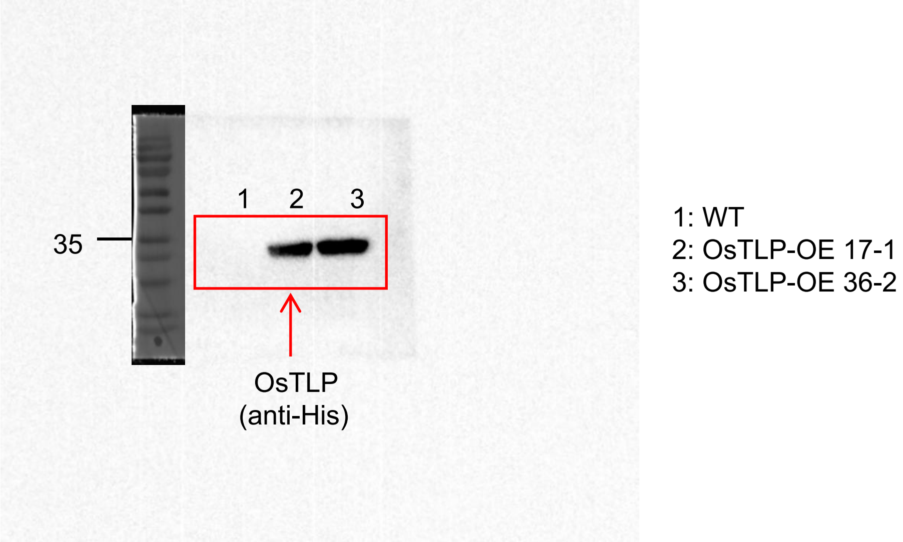

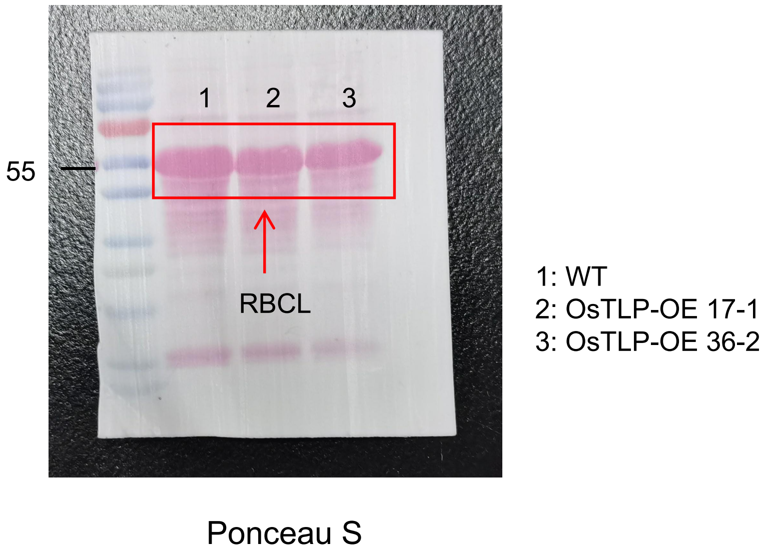

Figure3-supplement2-Source data 2. Original membranes corresponding to Figure 3, supplement2. Rainbow molecular weight markers were employed. Western blot of the target protein (top) and Ponceau S staining (bottom). RBCL as loading control. lane 1: WT; lane 2: OsTLP-OE 17-1; lane 3: OsTLP-OE 36-2. The antibodies used for detection are indicated on the figure.
